# Targeting Siglec-engaging immunosuppressive sialoglycans to suppress prostate cancer bone metastasis

**DOI:** 10.1038/s41416-026-03544-5

**Published:** 2026-07-13

**Authors:** Ziqian Peng, Kirsty Hodgson, Matthew Fisher, Shenglin Mei, Margarita Orozco-Moreno, Lizhi Cao, Michael Kemp, Wayne Gatlin, Louisa Donald, Libby Blencoe, Feier Zeng, Michelle A. Lawson, Daniel Ungar, David B. Sykes, David J. Elliott, Li Peng, Benjamin Schumann, Richard Beatson, Ning Wang, Jennifer Munkley

**Affiliations:** 1 Newcastle University Centre for Cancer, Newcastle University Institute of Biosciences, Newcastle, UK; 2 The Mellanby Centre for Musculoskeletal Research, Division of Clinical Medicine, School of Medicine and Population Health, University of Sheffield, Sheffield, UK; 3 Fralin Biomedical Research Institute, Virginia Tech FBRI Cancer Research Center, Washington, DC USA; 4 Department of Biomedical Sciences and Pathobiology, College of Veterinary Medicine, Virginia Tech, Blacksburg, VA USA; 5 Palleon Pharmaceuticals, Waltham, MA USA; 6 Leicester Cancer Research Centre, School of Medical Sciences, University of Leicester, Leicester, UK; 7 Department of Biology, University of York, York, UK; 8 Massachusetts General Hospital Cancer Center, Boston, MA USA; 9 The Chemical Glycobiology Laboratory, The Francis Crick Institute, London, UK; 10 Department of Chemistry, Imperial College London, London, UK; 11 Faculty of Chemistry and Food Chemistry, TUD Dresden University of Technology, Dresden, Germany; 12 School of Cancer & Pharmaceutical Sciences, King’s College London, London, UK

**Keywords:** Bone metastases, Prostate cancer

## Abstract

**Background:**

Prostate cancer is a leading cause of male cancer-related deaths over the age of 50. New treatment options are urgently needed, especially for prostate tumours that have spread to bone. Targeting aberrant sialylation holds substantial potential for the development of new therapeutics but is relatively unexplored in the context of prostate cancer.

**Methods:**

Here, using high-affinity Siglec-based sialoglycan-binding reagents (HYDRA) for immunohistochemistry, we quantify tumour sialoglycans in tissues representing the full clinical heterogeneity of prostate cancer. Using immunofluorescence, we profile Siglec receptors in prostate tumours, and using syngeneic mouse models, we evaluate an engineered dual-action sialidase (E-612) as a treatment for prostate cancer bone metastasis.

**Results:**

We find that sialoglycans that can engage Siglec-3 (CD33), Siglec-7 and Siglec-9 are upregulated in bone metastatic prostate cancer and show that sialoglycan ligands for Siglec-7 and -9 correlate with poorer patient prognosis. Furthermore, we reveal Siglec receptors and Siglec ligands are co-expressed by immune cells in prostate-derived tumours growing in bone. Indicating the Siglec-sialoglycan axis is clinically actionable in prostate cancer, we show systemic therapy with E-612 can suppress the growth of tumours, increase immune cell infiltration and prolong survival times of mice with bone metastasis.

**Conclusions:**

Our findings identify a novel mechanism involving Siglec-engaging sialoglycans in driving the growth of prostate cancer bone metastasis and demonstrate that targeting this axis using an engineered sialidase can impede lethal prostate cancer progression.

**Figure:**
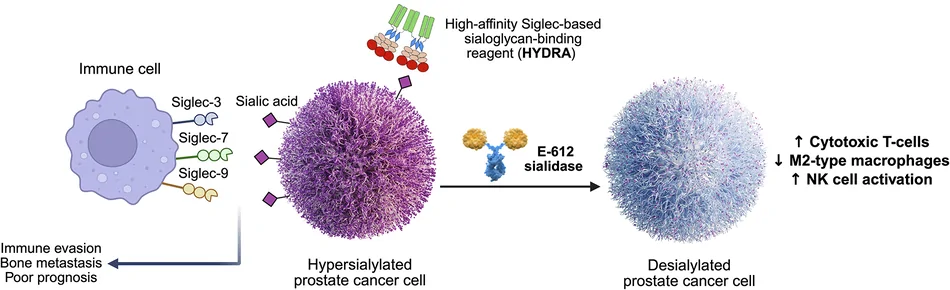

## Introduction

Prostate cancer is a common cancer in men leading to more than 350,000 deaths worldwide every year [1]. When prostate cancer is localised, curative therapies are available and the 5-year survival rate is 97%, but for patients with metastasis, 5-year survival can be as low as 32% [2]. The growth of prostate tumours is driven by androgen receptor (AR) signalling, and initial therapeutic options for advanced prostate cancer are hormone-based therapies such as anti-androgens [3–5]. However, eventually most tumours will become resistant to hormone therapy and progress to lethal castrate resistant prostate cancer (CRPC) [6]. Bone metastasis is common in prostate cancer, with up to 80% of patients with advanced disease diagnosed with tumours that have spread to the skeleton [2]. Although it is possible to manage the symptoms [7], no curative treatments are available for prostate cancer that has spread to bone and there is a critical need to develop new therapeutic options.

An area of innovation in the search for new cancer therapies is the targeting of cancer-associated glycosylation [8, 9]. Glycans decorate all cells and most secreted proteins and play essential roles in all biological processes [10]. Cancer cells have a different ‘glycan coat’ than healthy cells [11, 12], and this aberrant glycosylation plays a role in all of the cancer hallmarks [12]. A common feature of tumours is an increased density of glycan structures terminating in sialic acid (sialoglycans), including truncated *O*-glycans, as well as an overexpression of tri- and tetra-antennary sialylated *N*-glycans [13, 14]. Altered sialylation can promote tumour cell survival and metastasis, immune evasion and drug resistance [13, 15, 16]. Sialoglycans can inhibit apoptosis by modifying cell surface receptors, including Fas and TNFR1, through preventing the formation of death-inducing signalling complexes [17, 18]. Aberrant sialylation also contributes to metastasis by promoting cell detachment and driving the spread of malignant cells, for example by modulating EGFR signalling [19] or the EMT pathway [20]. Sialoglycan-covered antigens can also act as ligands for receptors called Siglecs (sialic acid–binding immunoglobulin-like lectins) that are expressed on immune cells, leading to immunosuppressive signalling that promotes immune evasion [16]. Humans express 14 unique Siglecs that have distinct preferred sialoglycan ligands [21]. The role of Siglecs in cancer appears to be diverse and complex, with the tumour glyco-code consisting of a heterogenous mixture of sialoglycans binding to Siglecs via both low affinity and high avidity interactions [22]. Since sialoglycans can fuel cancer progression, they are attractive targets for novel cancer therapies. Strategies to block aberrant sialylation in tumours, including sialylation inhibitors, Siglec blocking antibodies and targeted sialidases, are being actively pursued [13].

The prostate cancer glycome, which includes all glycans synthesised by cells, has remained relatively understudied. However, glycans hold great potential as biomarkers and therapeutic targets [23–25]. Studies have functionally linked altered sialylation to prostate cancer growth, bone metastasis and anti-tumour immunity [26–28] and provided proof of principle data demonstrating the potential to target sialylated glycans to impede prostate cancer progression [27, 29]. ST6GAL1-mediated α2,6 sialylation of *N*-glycans can drive prostate cancer growth and promote the spread of tumours to bone [26, 27], and ST3GAL1, which adds sialic acid residues in α2,3 linkage to *O*-glycans, can synthesise Siglec ligands and mediate anti-tumour immunity in prostate cancer [28]. *N*-glycan mass spectrometry imaging [30] has identified significant changes to the glycosylation patterns in various stages of prostate cancer, including an upregulation of α2,3 and α2,6 linked tetra-antennary *N*-glycans in advanced disease [26, 27, 31, 32]. Emerging research suggests that sialoglycans with the capacity to engage Siglec receptors may be upregulated on the surface of prostate cancer cells [22, 28, 33]. However, a correlation between sialoglycans that can engage Siglec receptors and prostate cancer progression has not yet been reported. Using Siglecs to reliably assess Siglec-engaging immunosuppressive sialoglycans is challenging due to the low Siglec to ligand binding affinity [21]. Therefore, comprehensive studies are needed that reliably monitor the expression of these ligands across the full heterogeneity of clinical prostate cancer tissues. Previous studies show that targeting the ligands for Siglec-7/9, by knockout of the glycosyltransferase ST3GAL1 or by using Siglec-7/9 blocking antibodies, can decrease prostate tumour growth and enhance anti-tumour immunity [28, 33]. We have previously shown that pre-treatment of prostate cancer cells with a sialyltransferase inhibitor removes sialylated glycans and can suppress bone metastasis [27]. These findings raise the possibility that systemic therapies to disrupt the Siglec-sialoglycan axis will likely be effective against bone metastatic prostate cancer. Several studies have reported that the targeted removal of Siglec ligands in the tumour microenvironment using sialidases can enhance anti-tumour immunity and halt tumour progression [34–37]. However, a sialidase therapy has not yet been evaluated in the context of prostate cancer or specifically for cancer that has spread to bone.

Here, using proprietary sialoglycan binding reagents, we monitored immunosuppressive sialoglycans in 7 well annotated prostate cancer tissue microarrays (TMAs), allowing us to investigate Siglec sialoglycan ligand expression across the full clinical heterogeneity of clinical prostate tumour tissues. Sialoglycan binding reagents provided by Palleon Pharmaceuticals consist of multimeric Siglec domains to achieve superior avidity [38]. Our findings reveal a significant upregulation of immunosuppressive sialoglycans, including ligands for Siglec-3 (CD33), Siglec-7 and Siglec-9, in primary prostate cancer tissues relative to matched control samples and in therapy resistant prostate tumours growing in bone. We show that sialoglycan ligands correlate with bone metastasis and that ligands for Siglec-7 and -9 are linked to reduced survival times in prostate cancer patients. We also reveal that Siglec receptors and Siglec ligands are co-expressed in the bone metastatic prostate tumour immune microenvironment (TIME). Furthermore, we demonstrate that an engineered dual-action sialidase (E-612), that strips Siglec ligands from prostate cancer cells, can increase immune cell infiltration and prolong survival times of mice with prostate cancer bone metastasis. Our study identifies a novel mechanism involving Siglec-engaging sialoglycans driving the growth of bone metastatic prostate tumours and indicates the Siglec-sialoglycan axis can be clinically targeted to impede prostate cancer progression.

## Methods

### Clinical cohorts

#### TMA cohort 1

A 40 case human prostate cancer TMA was purchased from Novus Bio (NBP2-30169).

#### TMA cohort 2

A previously published 96 case TMA comprising duplicate cores of normal prostate tissue and prostate adenocarcinoma of different Gleason grades was purchased from US Biomax (PR1921b) [39, 40].

#### TMA cohort 3

A previously published TMA consisting of prostatectomy tissue from 200 cases of prostate cancer and matched normal tissue (four cores each) [26, 39].

#### TMA cohort 4

A previously published TMA consisting of prostatectomy tissue from 200 cases of untreated primary prostate cancer and 10 cases of rapid autopsy FFPE tissue samples from prostate-derived tumours growing in bone [27].

#### TMA cohort 5

174 case TMA with untreated (hormone naïve) and treatment resistant (CRPC) tissues constructed as part of the Movember Global Action Plan 1 Unique tissue microarray (GAP1-UTMA) project [41].

#### TMA cohort 6

100 case CRPC metastatic clinical heterogeneity TMA intended to assess the heterogeneity of molecular markers across different anatomic sites in lethal prostate cancer metastasis. This TMA has been previously published [42] and was created as part of the Movember Global Action Plan 1 Unique tissue microarray (GAP1-UTMA) project [41].

#### TMA cohort 7

A 100 case prostate cancer and normal tissue TMA with survival data was purchased from US Biomax (MPR1005sur) [43].

#### Primary prostate cancer tissue

Patient tissue samples were collected with ethical permission from Castle Hill Hospital (Cottingham, Hull) (Ethics Number: 07/H1304/121). Use of patient tissue was approved by the Local Research Ethics Committees. Patients gave informed consent and all patient samples were anonymised.

#### Bone metastasis tissue samples from rapid autopsy

20 cases of rapid autopsy FFPE tissue samples from prostate-derived tumours growing in bone were kindly provided by Dr. Colm Morrissey (University of Washington) via the Prostate Cancer Biorepository Network (PCBN). Biopsies of metastatic bone sites were obtained from patients with CRPC within hours of death using a cordless drilling trephine (DeWalt Industrial Tool) and model 2422-51-000 trephine (DePuy). Bone cores were fixed in 10% neutral buffered formalin, decalcified with 10% formic acid and paraffin embedded.

### Immunohistochemistry

The immunohistochemistry (IHC) protocol using the HYDRA reagents was adapted from previously published methodology [22]. HYDRA-3, HYDRA-7 and HYDRA-9 reagents, which correspond to ligand binding domains of their corresponding Siglecs (Siglec-3 (CD33), Siglec-7 and Siglec-9 respectively) were provided by Palleon Pharmaceuticals (Waltham, MA). HYDRA reagents were diluted in Antibody Diluent (Dako, S302283-2). All slides were stained using the IHC Prep & Detect Kit for Rabbit/Mouse Primary Antibody (Proteintech, PK10019), following the manufacturer’s protocols. A Sodium Citrate Antigen Retrieval Buffer (Proteintech, PR30001) was used, and tissues were incubated with  HYDRA-3 at 4.5 μg/mL, HYDRA-7 at 1.5 μg/mL or HYDRA-9 at 0.25 μg/mL for 1 h before the secondary antibody incubation. HistoClear (National Diagnostics, HS-200) and HistoMount (National Diagnostics, HS-103) were used instead of Xylene and the mounting media from the kit. The TMAs were scored by a pathologist using the 0–300 HistoScore method [44, 45]. Only epithelial cells were scored. Images were acquired and processed using the ZEISS Axioscan 7 and ZEN Microscopy Software (blue edition).

### Analysis of single cell RNAseq data

Primary prostate cancer and prostate cancer bone metastasis single cell data were downloaded from GSE143791 [46] and GSE181294 [47]. Normalised gene expression matrixes and the cell annotations were utilised. Visualisation of the single-cell RNAseq data was performed using the Seurat package (v 5.3.0) in RStudio. Standard Seurat workflows were used for visualisation of selected marker gene expression using the DotPlot function.

### Cell culture

CWR22RV1 (RRID:CVCL_1045) and RM1 cells (RRID:CVCL_B459) were cultured as described previously [27, 48]. CWR22Rv1 GNE-knockout cells were generated using GLCNE CRISPR/Cas9-KO (sc-406100) and HDR (sc-406100-HDR) plasmids from Santa Cruz Biotechnology. Control cells were generated using Control CRISPR/Cas9 plasmid (sc-418922). Plasmids were transfected with Lipofectamine 3000 Transfection Reagent (ThermoFisher Scientific, L3000008) following the manufacturer’s protocol. Knockout cell clones were selected with puromycin (2.5 µg/mL) and grown in media supplemented with 5 mM N-Acetyl-D-mannosamine (ManNAc) (Merck Life Science, A8176) to restore sialylation. GNE knockout was validated by immunoblot. All of the cell lines used were validated using STR profiling and tested monthly for mycoplasma contamination.

### Immunoblotting

Protein was extracted with 8 M urea lysis buffer. Lysates (25 µg) were subjected to SDS PAGE followed by western blot with primary antibodies diluted at 1:2000 for GNE Rabbit Monoclonal Antibody (Abcam, ab189927) and 1:50000 for GAPDH Mouse Monoclonal Antibody (Proteintech, 60004-1-Ig) incubated at 4 °C overnight. Fluorescent secondary antibodies: anti-rabbit IgG (H + L) DyLight® 800 4X PEG Conjugate (Cell Signaling Technology, 5151P) and anti-mouse IgG (H + L) DyLight® 680 Conjugate (Cell Signaling Technology, 5470P) both diluted at 1:5000, were incubated for 1 h at room temperature and bands were detected with the Odyssey Fc Imaging System (LI-COR).

### Immunofluorescence (clinical tissues and cell lines)

To monitor sialylation changes in cell lines following E-612 treatment, cells were cultured in Nunc™ Cell-Culture Treated 4-well dishes (Thermo Scientific, 176740) in complete media and treated with E-612 (Palleon Pharmaceuticals, Waltham, MA) at the concentrations indicated for 24 h at 37 °C in a humidified incubator with 5% carbon dioxide. Next, cells were washed with PBS before permeabilization and fixation with ice-cold absolute methanol for 10 min at −20 °C. After another PBS wash, cells were blocked with 1X Carbo-Free™ Blocking Solution (1X CFB) (Vector Laboratories, SP-5040-125) for 1 h at room temperature, before they were incubated overnight in the dark at 4 °C in 1:1000 Fluorescein-labelled Peanut Agglutinin (PNA) Lectin (Vector Laboratories, FL-1071), 1:1000 Fluorescein-labelled Erythrina Cristagalli Lectin (ECL, ECA) (Vector Laboratories, FL-1141-5), 1:1000 Fluorescein-labelled Concanavalin A (Con A) Lectin (Vector Laboratories, FL-1001-25) or 1:400 SureLight® 488-conjugated SiaFind Alpha 2,3-Specific Lectenz® (Lectenz Bio, SK2301F). Finally, following PBS washes, cells were stained with 1:1200 Hoechst Nucleic Acid Stain (Thermo Scientific, 62249) for 15 min at room temperature. Cells were then washed with PBS and mounted using ProLong™ Gold Antifade Mountant (Thermo Fisher, P36930).

For Siglec dual staining in clinical tissues, FFPE-fixed slides were dewaxed and rehydrated before antigen retrieval at 95 °C for 20 min using a Sodium Citrate Antigen Retrieval Buffer (Proteintech, PR30001). Tissues were then blocked with 1X CFB for 1 h at room temperature, following overnight incubation in the dark at 4 °C in 1:100 CD33 Rabbit Monoclonal Antibody [EPR23051-101] (Abcam, ab269456), 1:200 Siglec-7/CD328 Polyclonal Antibody (Proteintech, 13939-1-AP), 1:200 Siglec-9 Polyclonal Antibody (Proteintech, 13377-1-AP), 1:200 HYDRA-3 (Palleon Pharmaceuticals), 1:200 HYDRA-7 (Palleon Pharmaceuticals), 1:500 HYDRA-9 (Palleon Pharmaceuticals), 1:50 Human NCR-1/NKp46 Mouse Monoclonal Antibody (R&D Systems, MAB1850), 1:200 CD14 Rabbit Polyclonal Antibody (Proteintech, 17000-1-AP), 1:200 CD14 Mouse Monoclonal Antibody (Proteintech, 60253-1-Ig), 1:500 AMACR/p504S Rabbit Polyclonal Antibody (Proteintech, 15918-1-AP), 1:200 AMACR Mouse Monoclonal Antibody [UMAB68] (UltraMAB, UM870012), 1:200 CD206 Rabbit Polyclonal Antibody (Proteintech, 18704-1-AP), 1:200 CD206 Mouse Monoclonal Antibody (Proteintech, 60143-1-Ig), 1:200 Cathepsin K Rabbit Polyclonal Antibody (Proteintech, 11239-1-AP) or 1:100 Pan Cytokeratin Monoclonal Antibody (AE1/AE3), Alexa Fluor™ 488-conjugated (Invitrogen 53-9003-80). Next, tissues were washed with PBS before 1 h incubation in the dark at room temperature in 1:1000 Goat Anti-Rabbit IgG H&L (Alexa Fluor® 594) (Abcam, ab150080), 1:1000 Goat Anti-Mouse IgG H&L (Alexa Fluor® 488) (Abcam, ab150113), or 1:500 Multi-rAb® CoraLite® Plus 594-Goat Anti-Rabbit Recombinant Secondary Antibody (H + L) (Proteintech, RGAR004), 1:100 Fluorescein (FITC)–conjugated Donkey Anti-Goat IgG(H + L) (Proteintech, SA00003-3), and 1:500 ChromoTek Nano-Secondary® anti-mouse IgG2a, recombinant VHH, CoraLite® Plus 488 [CTK0114] (Proteintech, smsG2aCL488-1). Finally, following PBS washes, tissues were stained with 1:1200 Hoechst Nucleic Acid Stain (Thermo Scientific, 62249) for 15 min at room temperature. Tissues were then washed with PBS and mounted using ProLong™ Gold Antifade Mountant (Thermo Fisher, P36930). Images were acquired and processed with the ZEISS Axio Imager 4 and ZEN Microscopy Software (blue edition).

To monitor changes in sialylation and immune cell expression following E-612 treatment in vivo, mouse tumour tissues (fixed using 10% neutral buffered formalin) were prepared as above (Siglec dual staining methods), before overnight incubation in the dark at 4 °C in 1:500 PNA Lectin (Vector Laboratories, FL-1071), 1:200 CD206 Rabbit Polyclonal Antibody (Proteintech, 18704-1-AP), 1:200 CD14 Rabbit Polyclonal Antibody (Proteintech, 17000-1-AP), 1:200 CD45 Rabbit Polyclonal Antibody (Proteintech, 20103-1-AP), 1:200 CD3 Rabbit Monoclonal Antibody [SP162] (Abcam, ab135372), 1:200 CD4 Rat Monoclonal Antibody [RM4-5] (Invitrogen, 14-0042-82), 1:200 CD8a Rat Monoclonal Antibody [53-6.7] (Invitrogen, 14-0081-82), 1:500 Mouse NCR1/NKp46 Rabbit Monoclonal Antibody [EPR23097-35] (Abcam, ab233558) and 1:50 Mouse Siglec-E Goat Polyclonal Antibody (R&D Systems, AF5806). Next, tissues were washed with PBS before 1 h incubation in the dark at room temperature in 1:1000 Goat Anti-Rabbit IgG H&L (Alexa Fluor® 594) (Abcam, ab150080) and 1:100 FITC–conjugated Donkey Anti-Goat IgG(H + L) (Proteintech, SA00003-3). Samples were then stained for nucleic acids and mounted as above.

### Expression and purification of engineered human Neu2 sialidase-Fc fusion (E-612)

A mutant of human Neu2 sialidase, incorporating structure-guided stabilising mutations, fused to human IgG1 Fc fragment, was designated E-612. DNA encoded E-612 was transfected into a CHO-K1 stable cell pool for recombinant protein expression (WuXi Biologics, Shanghai, China). Protein expression was conducted at 3 litre scale and cultured for 15 days following standard cell culture processes. E-612 was purified from cell culture supernatants using a 3-step process: Protein-A chromatography, followed by CHT (ceramic hydroxyapatite type II) chromatography and lastly SEC (size exclusion chromatography). CHT chromatography used the following process parameters, binding in CHT-A buffer (30 mM Phosphate at pH6.5, 10 mM MES pH6.5, 0.1 mM CaCl_2_), and gradient elution with 35% CHT-B buffer (30 mM Phosphate at pH6.5, 100 mM MES pH6.5, 0.1 mM CaCl_2_, 1 M NaCl). Final protein was buffer exchanged into the formulation buffer (137 mM NaCl, 2.7 mM KCl, 10 mM Na_2_HPO_4_, 1.8 mM KH_2_PO_4_, pH 7.4, 25 mM Na Citrate, 1 mM CaCl_2_, 10% (w/v) Glycerol).

### Flow cytometry

Cells were cultured in 96-well plates (Sarstedt, 83.3924) in complete media and treated with E-612 (Palleon Pharmaceuticals, Waltham, MA) for 24 h at 37 °C in a humidified incubator with 5% carbon dioxide. Cells were then washed with PBS, trypsinised and centrifuged at 500 × *g* for 5 min at room temperature. They were washed twice with 1X CFB before being resuspended in 100 μL of 1:2000 Fluorescein-labelled Peanut Agglutinin (PNA) Lectin (Vector Laboratories, FL-1071), 1:2000 Fluorescein-labelled Erythrina Cristagalli Lectin (ECL, ECA) (Vector Laboratories, FL-1141-5) or 1:400 SureLight® 488-conjugated SiaFind Alpha 2,3-Specific Lectenz® (Lectenz Bio, SK2301F) in 1X CFB and incubated for 30 min in the dark at 4 °C. Next, cells were washed with PBS twice before being resuspended in 500 μL of PBS with 1 μg/mL propidium iodide. Finally, cells were run on a BD LSRFortessa™ Cell Analyzer (BD Biosciences) with 10,000 events recorded per sample. Data was analysed using FCS Express™ 7 Plus version no. 7.14.0020.

### Murine white blood cell fixation and flow cytometry

200 μl blood was collected from the RM1 intra-caudal study mice from terminal cardiac bleeding using a 25 G needle and 1 ml syringe and added to 30 μl of 0.5 μM EDTA in 2 ml collection tubes. Red blood cells were lysed using 1X Red Blood Cell (RBC) Lysis Buffer (Invitrogen, 00-4333-57). Pellets were washed twice with 1 ml 1X PBS then fixed with 1% PFA on ice for 10 min. After two washes, cells were resuspended in 1X PBS with 1% v/v BSA and stored at 4 °C. For flow cytometry, pellets were stained with 100 μl 2.5 μg/ml Fluorescein labelled Peanut Agglutinin (PNA) lectin (Vector Laboratories, FL-1071-10) diluted in 1X CFB for 30 min at 4 °C in the dark. After two washes with FACS buffer (1X PBS with 1% v/v FBS), cells were stained with 100 μl 0.25 μg/ml CD45-PE antibody (BioLegend, 103105) diluted in FACS buffer for 15 min at 4 °C in the dark. Cells were washed twice then resuspended in 500 μl FACS buffer. Data was acquired on a BD LSRFortessa™ Cell Analyzer (BD Biosciences) with 10,000 events recorded per sample. Cells were identified by size and granularity (FSC-A vs. SSC-A) followed by gating on CD45 (561 586/15-A vs SSC-A) to isolate leucocytes. Finally, a histogram was used to determine the mean fluorescence intensity (MFI) for PNA (488 530-30-A vs Count). Data was analysed using FCS Express™ 7 Plus version no. 7.14.0020.

### NK cell cytotoxicity assay

CWR22vR1 cells were stained with CFSE (423801, Biolegend) as per manufacturer’s instructions. Labelled cells were plated out at 1 × 10^5^/ml in 24 well plates and allowed to adhere overnight, before being treated with 1500 nM E-612 or vehicle (control) for 4 h at 37 °C. During this time, primary NK cells were isolated from healthy donor leucocyte cones (NHS Blood and Transplant Service; NHSBTS) using CD56 microbeads (130-050-401, Miltenyi Biotec) and the MACS (Magnetic-activated cell sorting) system as per manufacturer’s instructions. NK cells (effectors; E) were added to the CWR22vR1 cells (targets; T) at an E:T ration of 10:1 and co-cultured for 18 h at 37 °C. After this, non-adherent cells plus supernatant were removed and spun down (400 × *g*/5 mins) with supernatant removed and stored at −20 °C for IFNγ ELISA (430101, Biolegend; assay carried out as per manufacturer’s instructions). Remaining adherent cells were removed using 5 mM EDTA and gentle pipetting and combined with the appropriate non-adherent cells. Cells were then stained with viability dye (L23102, ThermoFisher; as per manufacturer’s instructions) and MAL II lectin (B-1265-1, Vectorlabs; binds α2,3 linked sialic acid) to assess desialylation. Cells were assessed for viability using flow cytometry (BD Accuri C6 Plus flow cytometer and software), with CFSE being used to separate target cells (stained) from effectors (unstained).

### Mouse models

#### RM1 metastasis study

Six-week old male C57BL/6 mice (RRID:MGI:2159769) were purchased from Charles River (Kent, UK) and housed in a controlled environment in Optimice cages (Animal Care Systems, Colorado, USA) randomly located in the cage rack with a 12 h light/dark cycle at 22 °C with ad libitum water and 2018 Teklad Global 18% protein rodent diet containing 1.01% Calcium (Harlan Laboratories, UK). RM1 cells [49] were resuspended in PBS to 1 × 10^6^ cells/mL. Twenty mice received single-cell suspensions of 50,000 RM1 cells/100 μL PBS via intra-cardiac injection. Mice were randomised into two groups to receive intra-peritoneal injection (IP) of E-612 (10 mg/kg) or vehicle control twice weekly. Upon reaching humane endpoint, mice were euthanized to generate a survival curve over a 48-day period.

#### RM1 intra-caudal study

To more focus on the bone metastasis, we adopted the intra-caudal artery injection model that recapitulates the process of bone metastasis in the hindlimbs only [50]. Briefly, sixteen 6-week-old male C57BL/6 mice were inoculated with a single-cell suspension of 1 × 10^6^ RM1 cells/100 μL PBS via intra-caudal artery injection. Mice were randomised into two groups to receive IP injection of E-612 (10 mg/kg) or vehicle control twice weekly from day 1 post-tumour inoculation. Tumour progression was monitored weekly based on bioluminescence using the In Vivo Imaging System system for 2 weeks and the images of each hindlimb as experimental units were blinded and quantified for tumour burden. Mice were euthanized on day 15 post-tumour inoculation, through exsanguination under general anaesthesia, followed by cervical dislocation.

Our study exclusively examined male mice because the disease modelled is only relevant in males. All procedures complied with the UK Animals (Scientific Procedures) Act 1986 and were reviewed and approved by the local Research Ethics Committees of the University of Sheffield under Home Office project licence PP3267943 (Sheffield, UK).

### Statistical analyses

Statistical analyses were conducted using the GraphPad Prism software (version Prism 10). Data normality was assessed prior to analysis, and homogeneity of variance was evaluated using an *F*-test or Levene’s test, as appropriate. Statistical tests were applied according to distributional assumptions and the experimental design, as indicated in the text and figure legends. Data are presented as the mean of three independent samples ± standard error of the mean (SEM). Statistical significance is denoted as *p < 0.05, **p < 0.01, ***p < 0.001 and ****p < 0.0001.

**Fig. 1:**
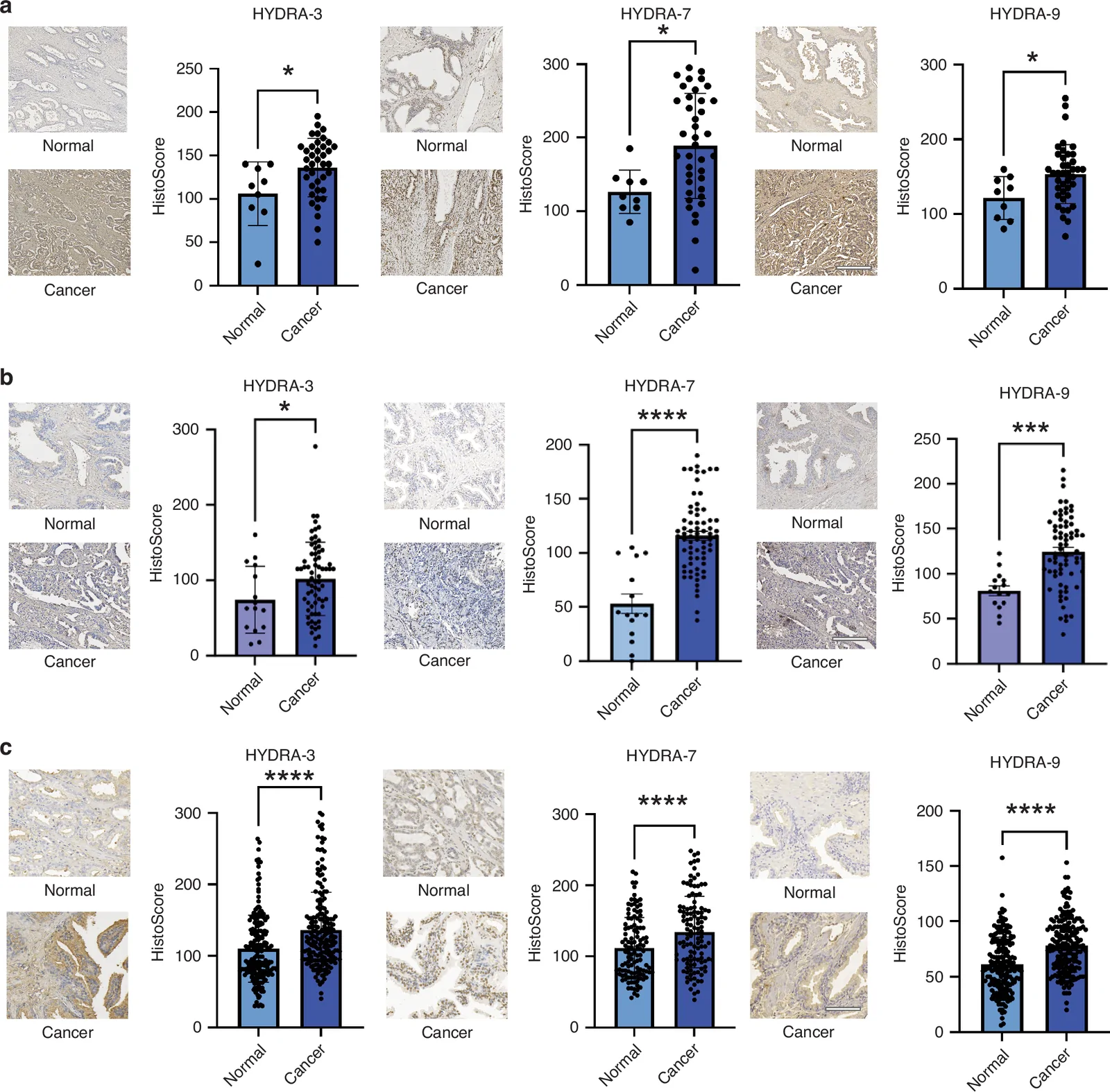
Prostate tumours have increased HYDRA scores compared to normal prostate tissue. **a** Analysis of Siglec-engaging sialoglycans using HYDRA immunohistochemistry in a 40 case TMA shows ligands for Siglec-3 (CD33) (unpaired *t* test, *p* = 0.0216), Siglec-7 (unpaired *t* test, *p* = 0.0143) and Siglec-9 (unpaired *t* test, *p* = 0.0271) are upregulated in prostate tumour tissue relative to normal prostate tissue. **b** Staining a previously published 96 case TMA [39, 82] containing 17 normal prostate tissue samples and 79 samples of prostate tumour tissue showed that sialoglycan ligands for Siglec-3 (unpaired *t* test, *p* = 0.0350), Siglec-7 (unpaired *t* test, *p* < 0.0001) and Siglec-9 (unpaired *t* test, *p* = 0.0009) are found at significantly higher levels in prostate tumours compared to normal prostate tissues. **c** HYDRA immunohistochemistry analysis of Siglec-3, -7 and -9 ligands in a previously published 200 case TMA [39, 82] containing four matched tumour and normal tissues from the same patient. Sialoglycan ligands recognised by Siglec-3 (paired *t* test, *p* < 0.0001), Siglec-7 (paired *t* test, *p* = 0.0001) and Siglec-9 (paired *t* test, *p* < 0.0001) are significantly increased in prostate cancer tissue relative to matched normal tissue from the same patient. Scale bar is 200 µm.

**Fig. 2:**
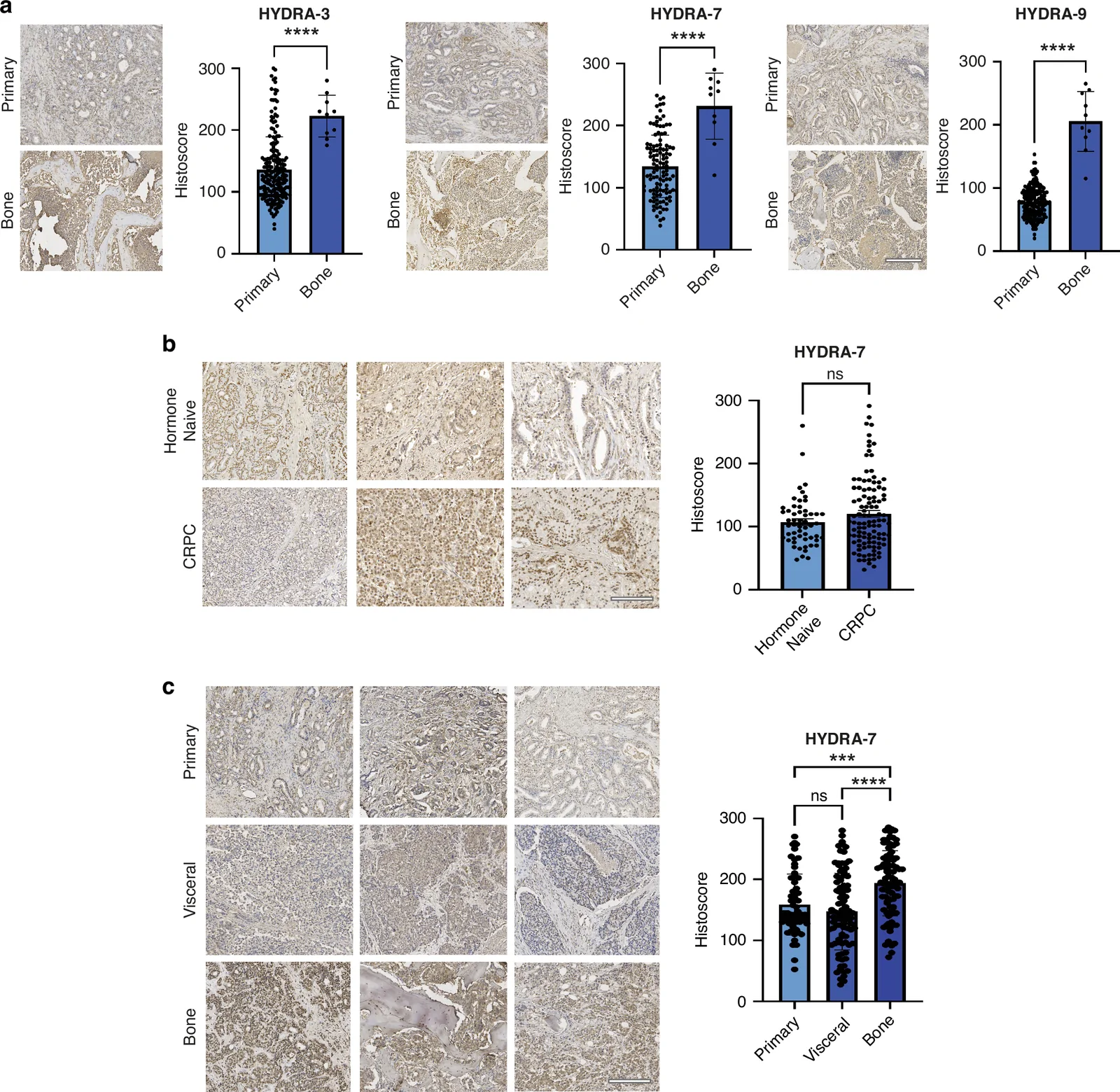
Siglec-engaging tumour immunosuppressive sialoglycans are upregulated in bone metastatic prostate tumours. **a** HYDRA immunohistochemistry analysis of ligands for Siglec-3 (CD33), Siglec-7 and Siglec-9 in untreated primary prostate tissue compared to metastatic castrate resistant prostate cancer (CRPC) growing in bone suggests all three sialoglycan ligands are increased in treatment resistant metastatic tumours relative to untreated primary prostate cancer tissues (*n* = 210, unpaired *t* tests, HYDRA-3 *p* < 0.0001, HYDRA-7 *p* < 0.0001, HYDRA-9 *p* < 0.0001). Scale bar is 200 µm. **b** Analysis of Siglec-7 ligands in a TMA generated by the Movember Global Action Plan 1 Unique tissue microarray (GAP1-UTMA) project [41]. HYDRA-7 immunohistochemistry shows Siglec-7 ligands are expressed at similar levels in untreated/hormone naïve primary prostate tumours compared to therapy resistant (CRPC) tissues (*n* = 174, unpaired *t* test, *p* = 0.0904). Scale bar is 200 µm. **c** HYDRA immunohistochemistry analysis of Siglec-7 ligands in a TMA containing primary prostate tissue and rapid autopsy tissue obtained from lethal visceral and bone metastatic tumours [42]. HYDRA-7 Histoscores were significantly higher in lethal bone metastatic prostate tumours compared to unmatched primary prostate tumours (*n* = 160, Welch’s ANOVA test, *p* < 0.0001). The levels of Siglec-7 ligands were significantly higher in prostate derived tumours growing in bone compared to matched visceral tumour tissue from the same patient (*n* = 100, paired *t* test, *p* < 0.0001). Scale bar is 200 µm.

**Fig. 3:**
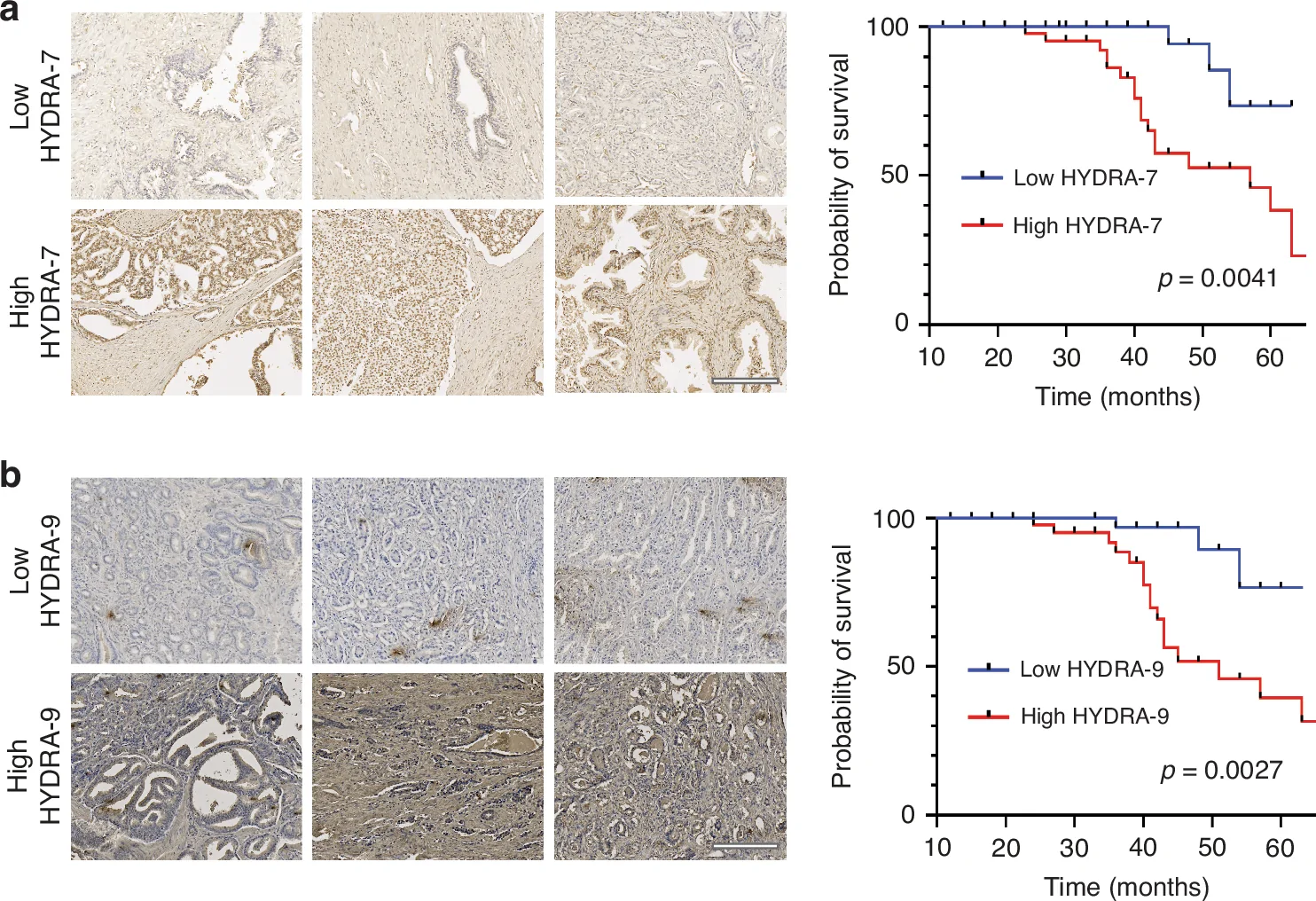
Sialoglycans that engage Siglec-7 and Siglec-9 correlate with poor patient survival times. **a** HYDRA-7 immunohistochemistry analysis of Siglec-7 ligands in a 100 case TMA. Stratification of patients based on high and low Siglec-7 ligand levels shows patients with high HYDRA-7 levels (defined as the top 50th percentile of expression) had significantly poorer survival rates compared to patients with low HYDRA-7 levels (defined as the bottom 50th percentile of expression) (*n* = 100, Kaplan-Meier regression model, *p* = 0.0041). **b** HYDRA-9 immunohistochemistry analysis of Siglec-9 ligands in a 100-case prostate cancer TMA. Stratification of patients based on high and low Siglec-9 ligand levels shows patients with high HYDRA-9 levels (defined as the top 50th percentile of expression) had significantly poorer survival rates compared to patients with low HYDRA-9 levels (defined as the bottom 50th percentile of expression) (*n* = 100, Kaplan-Meier regression model, *p* = 0.0027). Scale bar is 200 µm.

## Results

### Sialoglycans that can engage Siglec-3 (CD33), Siglec-7 and Siglec-9 are upregulated in prostate tumours

Previous studies suggest that sialoglycans with the capacity to engage Siglec-7 and Siglec-9 are upregulated on the surface of prostate cancer cells [22, 28, 33]. However, these findings were based on small sample sizes (<50 clinical tissues) and did not investigate correlations between sialoglycans that can engage Siglec receptors and prostate cancer progression. The Siglec-Fc proteins utilised to detect sialoglycans, while valuable tools, have reported limitations for ligand specificity and avidity [51]. To overcome this, we utilised the novel HYDRA reagents (Palleon Pharmaceuticals) including HYDRA-3, -7 and -9 (that consist of multimeric Siglec domains with higher avidity) to detect sialoglycans recognised by Siglec-3 (CD33), Siglec-7, and Siglec-9 respectively [22, 38]. Our study was focused on profiling Siglec-3, -7 and -9 sialoglycan ligand expression on prostate tumours, since among the 14 Siglecs in humans, these are the major inhibitory Siglecs on both innate and adaptive immune cells [22]. Using HYDRA-3, -7 and -9 immunohistochemistry, we monitored the levels of sialoglycans that can engage Siglec-3, -7 and -9 across three different tissue microarrays (TMAs). The HYDRA reagents have previously been validated for use in clinical sample testing, and have already been assessed for specificity, reproducibility and background/cross-reactivity. We confirmed specificity of our HYDRA immunohistochemistry assays for use on Formalin Fixed Paraffin Embedded (FFPE) prostate tissues by showing reduced binding to Siglec ligands in CWR22Rv1 cells with knockout of UDP-GlcNAc 2-epimerase/ManNAc kinase (GNE), a master regulator of sialic acid synthesis [52] (Supplementary Fig. 1). First, a TMA with tissue samples from 40 prostate cancer patients (TMA cohort 1) showed that all three sialoglycan types are expressed at significantly higher levels in prostate cancer tissue relative to normal prostate tissue (Fig. 1a). Second, analysis of a 96 case TMA containing 17 normal prostate tissue samples and 79 samples of prostate tumour tissue (TMA cohort 2) also showed that sialoglycan ligands for Siglec-3, -7 and -9 are significantly upregulated in prostate tumours (Fig. 1b). Finally, staining of a TMA comprising matched normal and prostate cancer tissue from 200 patients (TMA cohort 3) showed that sialoglycan ligands for Siglec-3 (paired *t* test, *p* < 0.0001), Siglec-7 (paired *t* test, *p* = 0.0001) and Siglec-9 (paired *t* test, *p* < 0.0001) are found at significantly higher levels in prostate cancer tissue relative to matched normal tissue from the same patient (Fig. 1c). Taken together, these novel molecular reagents reveal upregulation of sialoglycans that engage Siglec-3, -7 and -9 in clinical prostate cancer tissue.

### Expression of Siglec-engaging sialoglycans correlate with bone metastasis and reduced survival times in patients

We next monitored ligands for Siglec-3, -7 and -9 across five patient cohorts to investigate if immunosuppressive sialoglycans might also correlate with more aggressive prostate tumours. Analysis of the 96 case TMA cohort shown in Fig. 1b revealed higher expression of ligands for Siglec-7, and -9 in high grade (Gleason 4–5) tumours compared to lower grade (Gleason 1–3) tumours (Supplementary Fig. 2). We also used HYDRA immunohistochemistry to analyse sialoglycans in a TMA comparing untreated primary prostate tissue and metastatic CRPC growing in bone (TMA cohort 4). Our findings show that ligands for all three Siglecs are increased in treatment resistant tumours growing in bone relative to untreated primary prostate cancer (Fig. 2a). Next, we further analysed Siglec-7 ligands in two additional TMA cohorts constructed as part of the Movember Global Action Plan 1 Unique tissue microarray (GAP1-UTMA) project [41]. This analysis revealed Siglec-7 ligands are expressed at similar levels in untreated (hormone naive) primary prostate tumours compared to treatment resistant (CRPC) tissues (*n* = 161, unpaired *t* test, *p* = 0.0904) (TMA cohort 5) (Fig. 2b). Next, to assess the heterogeneity of Siglec-7 ligands across different anatomic sites in lethal prostate cancer metastasis, we analysed a 160 case TMA that contains primary prostate tissue and rapid autopsy tissue obtained from lethal visceral and bone metastatic tumours (TMA cohort 6). Analysis of this TMA confirmed upregulation of Siglec-7 ligands in prostate cancer tissue relative to matched normal tissue from the same patient (Supplementary Fig. 3) and further revealed significant upregulation of Siglec-7 engaging sialoglycans in bone metastatic prostate tumours (relative to unmatched primary prostate tissue or matched visceral tumours from the same patient) (Fig. 2c). As the Movember GAP1-UTMA project is no longer active, it was not possible for us to access additional GAP1-UTMA slides, meaning we have been unable to monitor ligands for Siglec-3 or -9 in these patient cohorts. Finally, to assess if the expression of Siglec ligands might correlate with survival times in prostate cancer patients, we analysed a previously published TMA comprising 100 cases of primary prostate tissues [43] (TMA cohort 7). Here, when we stratified prostate cancer patients based on high and low HYDRA staining levels, defined as the top and bottom 50th percentile of expression, patients with high levels of Siglec-7 ligands had significantly poorer survival rates compared to patients with low levels of Siglec-7 ligands. Patients with high levels of Siglec-9 ligands also had significantly reduced survival times compared to patients with low levels of Siglec-9 ligands. However, when comparing prostate cancer patients with high and low levels of Siglec-3 ligands, there was no significant difference in survival rates (Fig. 3 and Supplementary Fig. 4). In summary, these data show that the expression of sialoglycan ligands for Siglec-3, -7 and -9 correlates with prostate cancer bone metastasis and ligands that engage Siglec-7 and -9 also associate with poorer prognosis in patients with prostate cancer.

### Siglec receptors and Siglec ligands are co-expressed in the prostate cancer bone metastatic TIME

**Fig. 4:**
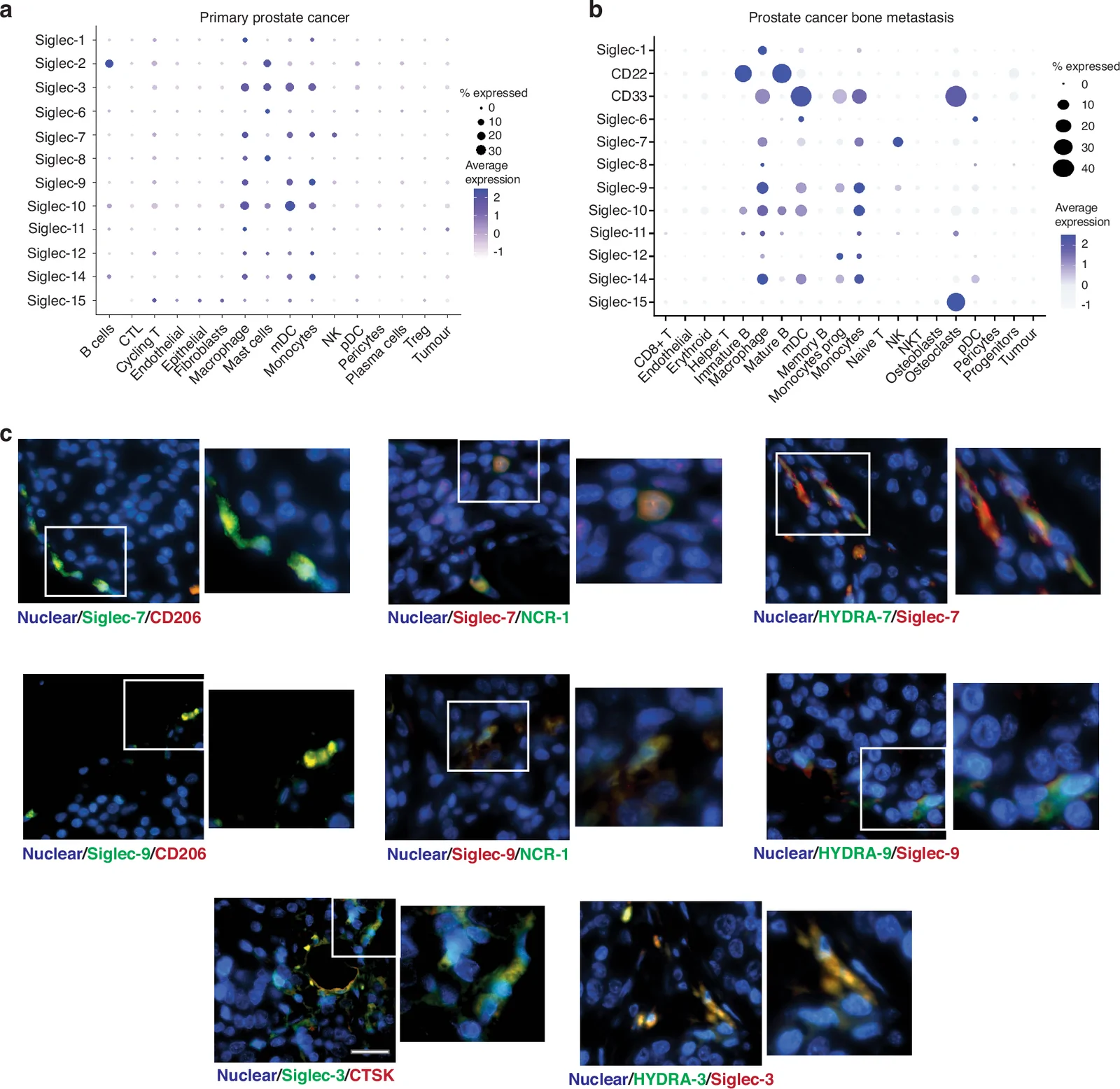
Siglec receptors and Siglec ligands are co-expressed in prostate-derived tumours growing in bone. Dot plots of *Siglec* gene expression levels at single cell resolution in prostate tumours using previously published single cell RNA-sequencing data from **a** primary prostate tumours [47] and **b** prostate cancer bone metastatic tissues [46]. **c** Dual immunofluorescence analysis of Siglec receptors, Siglec ligands and immune cell markers in prostate cancer bone metastasis tissue samples. This analysis was also carried out for primary prostate tissue (Supplementary Figs. 5 and 6). Siglec-7, and -9 are co-expressed with CD14 (a myeloid marker), CD206 (an M2 macrophage marker) and with NCR-1 (an NK cell marker) in bone metastatic prostate tumours. Siglec-3 (CD33) is co-expressed with cathepsin K (an osteoclast marker). Furthermore, Siglec-3 (CD33), Siglec-7 and Siglec-9 are co-expressed with their respective sialoglycan ligands (detected using HYDRA immunofluorescence) in bone metastatic prostate tumours. Scale bar is 20 µm.

Our findings above show that immunosuppressive sialoglycans are upregulated in prostate tumours and higher levels of these glycans correlate with metastasis and poorer patient prognosis. Recent findings show that Siglec-7 and -9 are mainly expressed by myeloid cells in both primary and bone metastatic prostate tumours [28, 33]. However, no comprehensive studies have been reported that integrate both Siglec-ligand staining with direct assessment of Siglec receptors on immune cells in the same tissues. Therefore, to monitor the corresponding patterns of Siglec expression at single cell resolution in the prostate cancer TIME, we analysed previously published single cell RNA-sequencing data from: (1) primary prostate tumours [47], and (2) prostate-derived tumour metastases growing in bone [46], and further validated our findings by performing dual immunofluorescence analysis of whole slide serial sections of independent clinical tissue. Our findings confirm Siglec-7 and -9 are expressed mainly on myeloid cells in clinical prostate cancer tissue and provide further insight into the expression of Siglec receptors in prostate tumours. We show that Siglec-7, and -9, are co-expressed with the myeloid marker CD14 and the M2 macrophage marker CD206 in bone metastatic tumours that also express immunosuppressive sialoglycans (Fig. 4 and Supplementary Figs. 5–8). Furthermore, we reveal that Siglec-7 and -9 are co-expressed with the NK cell marker NCR-1, and Siglec-3 is co-expressed with the osteoclast marker cathepsin K. Siglec receptors co-localised with HYDRA-3, -7 and -9, and with α-methylacyl-CoA racemase (AMACR), thus confirming their expression within cancerous tissues and with ligands for Siglec-3 (CD33), Siglec-7 and Siglec-9 within bone metastatic tissues. Together with the findings above, our data provides evidence of Siglec-engaging sialoglycans and their respective ligands and immunoreceptors in prostate-derived tumours growing in bone and suggests that Siglec biology may play a role in immune cell functions in both primary and bone metastatic prostate cancer.

### An engineered dual action sialidase (E-612) can effectively strip Siglec ligands from prostate cancer cells

**Fig. 5:**
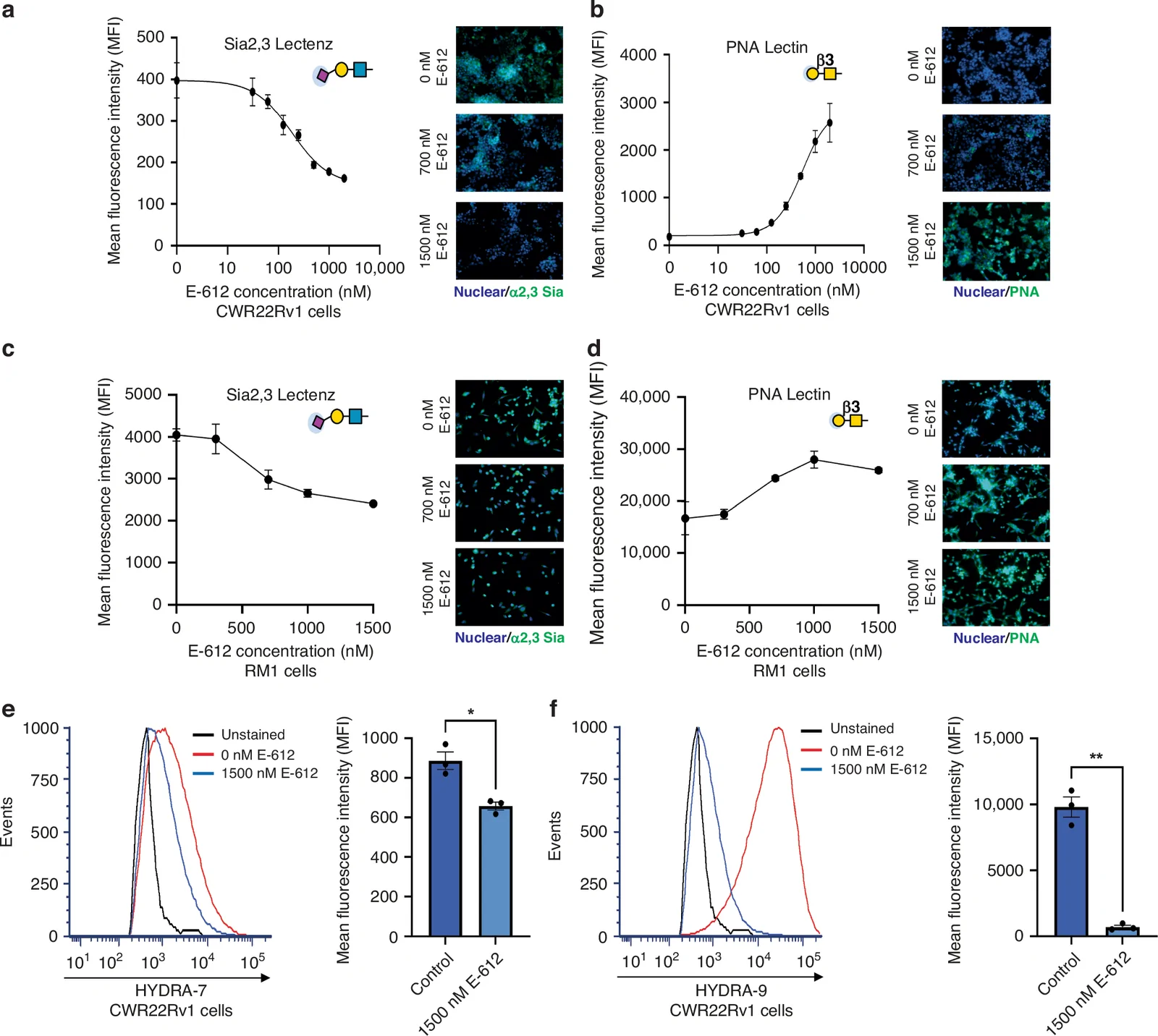
E-612 bisialidase can effectively strip Siglec ligands from prostate cancer cells. **a** Detection of α2-3 linked sialylated glycans in CWR22Rv1 cells using α2-3 Lectenz flow cytometry. CWR22Rv1 cells were treated with a range of concentrations of E-612 for 24 h. CWR22Rv1 cells treated with 1500 nM E-612 had reduced levels of α2-3 Lectenz binding indicating a reduction in α2-3 linked sialylation in these cells. Reduced levels of α2-3 linked sialylated glycans were also detected in cells treated with E-612 using α2-3 Lectenz immunofluorescence. **b** Lectin flow cytometry and lectin immunofluorescence assays show CWR22Rv1 cells treated with E-612 for 24 h have increased levels of binding to PNA lectin (which recognises galactosyl residues [55] uncovered by the removal of sialic acids). **c** Detection of α2-3 linked sialylated glycans in mouse RM1 prostate cancer cells using α2-3 Lectenz flow cytometry. RM1 cells were treated with a range of concentrations of E-612 for 24 h. RM1 cells treated with 1500 nM E-612 had reduced levels of α2-3 Lectenz binding indicating a reduction in α2-3 linked sialylation in these cells. Reduced levels of α2-3 linked sialylated glycans were also detected in cells treated with E-612 using α2-3 Lectenz immunofluorescence. **d** Lectin flow cytometry and lectin immunofluorescence assays show RM1 cells treated with E-612 for 24 h have increased levels of binding to PNA lectin (which recognises galactosyl residues [55] uncovered by the removal of sialic acids). **e** HYDRA-7 flow cytometry shows treatment with 1500 nM E-612 significantly reduces the binding of HYDRA-7 reagent to CWR22Rv1 cells indicating that E-612 has removed the sialoglycan ligands for Siglec-7 from the cell surface (unpaired *t* test, *p* = 0.0207) **f** HYDRA-9 flow cytometry shows treatment with 1500 nM E-612 significantly reduces the binding of HYDRA-9 reagent to CWR22Rv1 cells indicating that E-612 has removed the sialoglycan ligands for Siglec-9 from the cell surface (unpaired *t* test, *p* = 0.0058).

Our findings above correlate the expression of Siglecs on immune cells with Siglec-engaging sialoglycans on prostate cancer cells which associate with bone metastasis and reduced survival times in patients. We thus hypothesised that removing sialoglycans from prostate cancer cells could be a novel therapeutic option to treat advanced prostate cancer. To investigate this hypothesis, we evaluated the potential of a proprietary engineered bisialidase reagent termed E-612, developed by Palleon Pharmaceuticals for prostate cancer therapy. E-612 is the second iteration of Palleon’s E-602 sialidase [53], which is in Phase 2 clinical trials (NCT05259696) for patients with advanced cancers (not including prostate cancer). In clinical studies, E-602 is currently given as weekly intravenous injections. In 95 subjects treated to date, E-602 has been well tolerated at doses up to 30 mg/kg. E-612 and E-602 have similar pre-clinical pharmacokinetics with minor changes made to E-612 to further improve stability and manufacturability (Supplementary Fig. 9). We used flow cytometry and immunofluorescence to assess the impact of E-612 treatment on prostate cancer cell recognition by several lectins. On CWR22Rv1 and murine RM1 prostate cancer cells, E-612 treatment led to reduced staining with α-2,3 Lectenz [54] that recognises α-2,3 linked sialic acid residues (Fig. 5a, c). Concomitantly, E-612 treatment led to increased recognition by peanut agglutinin (PNA) (Fig. 5b, d) and Erythrina Cristagalli Lectin (ECL) (Supplementary Fig. 10A, B) that bind terminal galactose and lactose/LacNAc residues and therefore signify the loss of sialic acid [55]. Staining with concanavalin A (ConA) lectin revealed no difference in α-D-mannose content of E-612-treated samples and confirmed the sialic acid specificity of these changes (Supplementary Fig. 10C, D) [55]. Next, to further assess the capacity of E-612 to desialylate prostate cancer cells, we performed HYDRA-7 and HYDRA-9 flow cytometry analysis of CWR22Rv1 prostate cancer cells treated with E-612. Our findings reveal treatment with 1500 nM E-612 for 24 h can strip sialic acid from the surface of CWR22Rv1 prostate cancer cells to remove ligands for both Siglec-7 and Siglec-9 (Fig. 5e, f). Previous studies indicate that prostate cancer cell lines express higher levels of Siglec-9 ligands compared to Siglec-7 ligands [33], which could account for the difference in the fluorescence intensity for HYDRA-7 and –9 upon treatment with the same concentration of sialidase. Confirming specificity of our HYDRA flow cytometry assays, we detected a significant reduction in Siglec-7 and Siglec-9 ligands when prostate cancer cells were treated with 200 μM of the sialyltransferase inhibitor P-3FaX-Neu5Ac for 72 h (Supplementary Fig. 11). Together, these findings demonstrate that the bisialidase E-612 can effectively strip sialic acid from the surface of prostate cancer cells to remove immunosuppressive sialoglycans that are engaged by Siglec receptors.

### Therapeutic desialylation with E-612 reduces the growth of tumours and prolongs survival times of mice with prostate cancer bone metastasis

**Fig. 6:**
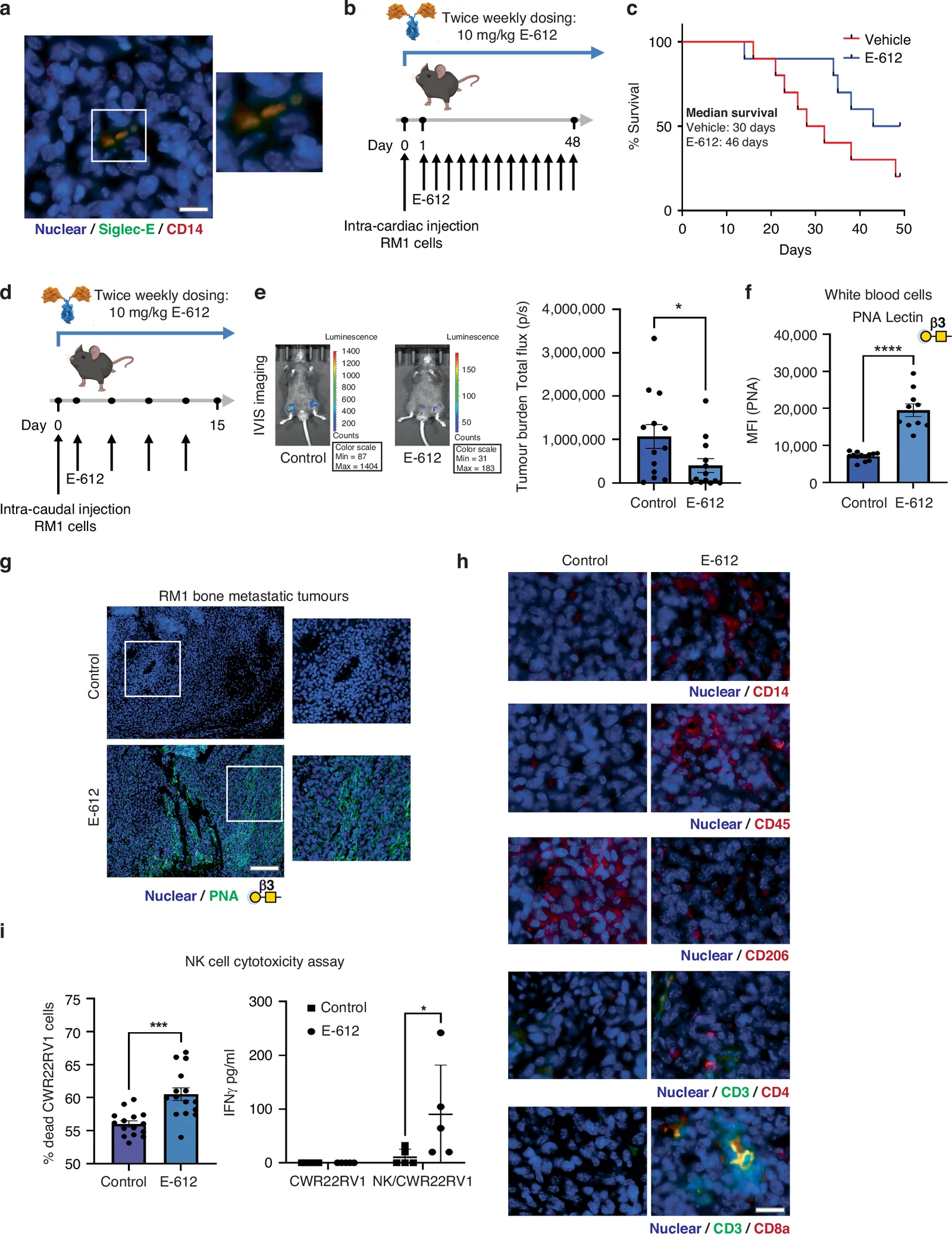
Desialylation of prostate cancer cells with E-612 reduces the growth of tumours, increases immune cell infiltration and prolongs survival times of mice with metastasis. **a** Dual immunofluorescence analysis of Siglec-E and CD14 (a myeloid marker) in RM1 prostate cancer tumours growing in mouse tibias. Siglec-E is co-localised with CD14 confirming its expression by myeloid cells within the murine prostate cancer TIME. Scale bar is 20 µm. **b** Using an RM1 intra-cardiac injection metastasis model, we investigated if therapeutic desialylation by E-612 can increase survival times of mice with metastatic prostate cancer, *n* = 10. **c** Twice weekly dosing with 10 mg/kg E-612 via intra-peritoneal injection prolongs the median survival rates of mice with metastatic prostate cancer by 53%. **d** Using an RM1 intra-caudal injection bone metastasis model [50], we tested if E-612 mediated tumour desialylation can suppress the growth of bone metastatic prostate tumours. **e** Systemic treatment with E-612 (twice weekly dosing with 10 mg/kg E-612 via intra-peritoneal injection) significantly reduces prostate cancer bone metastasis tumour burden (unpaired *t* test, *p* = 0.0456, *n* = 13). **f** PNA lectin flow cytometry analysis of white blood cells from mice treated with E-612. Systemic E-612 therapy significantly increases the levels PNA lectin binding to circulating immune cells (PNA lectin recognises galactosyl residues that are uncovered by the removal of sialic acids [55]) (Welch’s *t* test, *p* < 0.0001). **g** PNA lectin immunofluorescence analysis of RM1 tumours growing in mouse tibias. E-612 therapy increases the levels of PNA lectin binding to tumours indicating reduced levels of sialylation. Scale bar is 100 µm. **h** Analysis of immune cells in control and E-612 treated RM1 bone metastatic tumours using immunofluorescence. Scale bar is 20 µm. **i** NK cell cytotoxicity assay. Left, % of dead CFSE stained CWR22Rv1 prostate cancer cells after 4 h treatment with 1500 nM E-612 or control followed by 18 h co-culture with primary NK cells at a 10:1 effector:target ratio (unpaired *t*-test, *p* = 0.0059 or 0.0004, *n* = 5 biological, *n* = 3 technical). Right, Concentration of IFNγ in supernatant from treated/untreated CWR22Rv1 cells alone (2-way ANOVA, multiple comparisons, *p* = 0.0156, *n* = 5 biological, *n* = 3 technical).

Previous studies have shown that targeting the ligands for Siglec-7/9 by knockout of the glycosyltransferase ST3GAL1 or by using Siglec-7/9 blocking antibodies can decrease prostate tumour growth and enhance anti-tumour immunity [28, 33]. We have previously shown that pre-treatment of prostate cancer cells with a sialyltransferase inhibitor leads to a reduction of sialylated glycans and suppresses bone metastasis [27]. These findings raise the possibility that systemic therapies to disrupt the Siglec-sialoglycan axis will likely be effective against bone metastatic prostate cancer. However, this has not yet been tested using pre-clinical mouse models. As Siglec-E is the most broadly expressed inhibitory Siglec in mice [56] and has previously been identified as a mediator of the effects of therapeutic desialylation [34], we next tested if Siglec-E is expressed in the mouse bone metastatic TIME. Analysis of RM1 prostate tumours growing within the tibias of mice revealed that Siglec-E is expressed by myeloid cells within the bone TIME (Fig. 6a). This finding raised the possibility of targeting the sialoglycan-Siglec-E axis to treat bone metastatic prostate cancer in mice. Next, we utilised syngeneic mouse models to investigate whether desialylation using E-612 can be used therapeutically for metastatic prostate cancer. Consistent with this, using an RM1 intra-cardiac injection metastasis model, systemic treatment with E-612 dosed with 10 mg/kg twice per week via intra-peritoneal injection prolonged the median survival rates of mice with metastatic prostate cancer by 53% (Fig. 6b, c). Next, using an RM1 intra-caudal injection bone metastasis model, that recapitulates the process of bone metastasis [50], we tested if E-612 mediated tumour desialylation can suppress the growth of bone metastatic prostate tumours. Systemic treatment with E-612 significantly reduced the tumour burden of bone metastatic prostate cancer (unpaired *t* test, p = 0.0456) (Fig. 6d, e). In both these studies, the body weights of both treatment groups increased similarly over the course of the study, suggesting no signs of acute toxicity. To confirm that E-612 can effectively mediate desialylation in vivo, we used PNA lectin flow cytometry and immunofluorescence to monitor the levels of sialic acid on white blood cells and tumour tissues isolated from mice undergoing E-612 therapy. Our data revealed that systemic E-612 treatment removes sialic acid from both circulating immune cells and prostate cancer tumours growing in bone to effectively desialylate the tumour microenvironment (Fig. 6f, g). Suggesting that therapeutic desialylation can increase immune responses in prostate cancer, immunofluorescence analysis of immune cells in bone metastatic tumour tissues, revealed increased CD8+ T cell infiltration and reduced levels of M2 macrophages in tumours treated with E-612 (Fig. 6h). Consistent with this, using an human primary immune cell primary co-culture model, we further show that E-612 mediated therapeutic desialylation of prostate cancer cells can enhance NK cell cytotoxicity (Fig. 6i). These findings demonstrate the feasibility of using therapeutic in vivo desialylation to remodel the prostate cancer TIME and suppress the growth of bone metastatic prostate cancer.

## Discussion

Prostate cancer is a leading cause of male cancer-related deaths worldwide [57]. Although effective therapies for prostate cancer exist, resistance to therapy is very common and there is a continuing need to develop new treatment options, especially for prostate cancer that has spread to bone. Many studies have profiled the molecular changes correlating with prostate cancer progression and bone metastasis, with most research focused on changes to the genome, transcriptome, epigenome, proteome and metabolome. However, despite the functional link between aberrant glycosylation and the cancer hallmarks [12], and the prostate being an abundant secretor of glycoproteins [58], the glycome is relatively understudied in prostate cancer [59]. Novel cancer therapies targeting sialoglycans are currently being developed and trialled, and it is timely to investigate how prostate cancer patients could best benefit from these advances.

Our findings show Siglec-engaging sialoglycans are highly expressed in prostate tumours and particularly in prostate tumours that have spread to bone. We propose the interaction of these immunosuppressive sialoglycans with Siglec receptors on immune cells contributes to immune suppression in prostate cancer. Previous research has demonstrated that in prostate tumours Siglec-7 and Siglec-9 are highly expressed on myeloid cells, including macrophages [28, 33] and that binding of Siglec-7/9 to sialoglycans can suppress immune cell killing in prostate cancer [33]. However, how immune cells mediate prostate tumour immune evasion via Siglec-dependent pathways is poorly understood. Our study confirms the expression of Siglec-7/9 on CD206+ macrophages and further reveals Siglec-7/9 are also expressed on NK cells, alongside their respective sialoglycan ligands in the prostate cancer bone TIME. Macrophages are the most abundant immune cell types in prostate cancer and studies show CD206+ macrophages are linked to poor patient prognosis and are enriched in the bone TIME of prostate cancer [46, 60–62]. Therapies aimed at reducing M2 macrophage infiltration, or promoting macrophage repolarisation to pro-inflammatory M1 phenotypes, are being actively investigated for metastatic CRPC [63]. Interactions between Siglec-7 and -9 on NK cells and tumour sialoglycans can block NK cell-mediated killing of tumour cells [64, 65]. In prostate tumours, NK cell infiltration is linked to improved patient outcomes [66], and in lethal metastatic CRPC there is a reported loss of NK cell activity [67]. Taken together, our findings suggest immunosuppressive sialoglycans may inhibit NK cell mediated killing of prostate cancer cells and highlight therapeutic desialylation as a promising strategy to enhance tumour infiltrating NK-cell mediated activity in prostate cancer. It will be important for future studies aimed at targeting tumour-associated macrophages and/or NK cells in prostate cancer to consider Siglec-sialoglycan biology and how harnessing this axis can best be used to improve patient outcomes.

Targeting cancer glycosylation represents an exciting yet unexplored therapeutic strategy to reshape the prostate cancer immune landscape and augment an anti-tumour immune response. A recent breakthrough study demonstrated that therapeutic desialylation can reshape macrophage phenotypes to allow effective immune checkpoint blockade (ICB) [34]. ICB for the treatment of advanced prostate cancer is effective in only a minority of patients [68], and ongoing clinical trials are now focused on developing novel combination therapies aimed at sensitising prostate tumours to ICB [69–72]. Together with the literature, our findings suggest a clear role for tumour sialylation in dampening anti-tumour immune responses in bone metastatic prostate cancer. Excitingly, our study shows therapeutic desialylation by E-612 remodels the prostate cancer TIME, correlating with a shift in macrophage polarisation and an increased infiltration of CD8+ T cells. A limitation of our study is that while murine Siglec-E is a functionally equivalent orthologue of Siglec-9, and is recognised as the major inhibitory Siglec in mice, it is not a true homologue [34]. Therefore, due to differences in the Siglec-sialoglycan axis, the murine models used may not accurately replicate human immune response to tumours. In future pre-clinical evaluation studies, we plan to further evaluate the efficacy and feasibility of therapeutic desialylation using human immune cell co-culture assays, and within additional mouse models of prostate cancer, including in the context of Siglec-E knockout and immune cell depletion. Moving forward, we propose therapeutic desialylation in combination with ICB offers a promising novel therapeutic avenue for prostate cancer to explore. Furthermore, we envisage HYDRA reagents can be utilised to identify which prostate cancer patients will benefit from therapeutic desialylation, and/or to monitor how patients respond to therapy. In line with this, in future studies we plan to further investigate the potential to use

HYDRA-3, -7 and -9 both as independent prognostic markers and also as a combined biomarker panel.

Numerous studies have highlighted a role for aberrant sialylation in cancer therapy resistance, suggesting that targeting cancer-associated glycans may enhance responsiveness to cancer treatment [16, 34, 73, 74]. Hormone therapy remains the backbone of prostate cancer treatment. Emerging studies suggest that beyond inhibiting testosterone and prostate tumour growth, hormone therapy also modulates the prostate TIME [75, 76]. Recent findings suggest androgen deprivation therapy (ADT) can transform prostate cancers to a more immunologically active phenotype within days [75], suggesting prostate cancer patients might be more responsive to immunotherapy in the early castration period. Moving forward, we propose combining ADT with therapeutic desialylation may be a promising approach in for example the pre-surgical neoadjuvant setting. By the time metastatic CRPC develops, the TIME is reported to be devoid of a significant immune infiltrate, with a predominance of suppressive myeloid cells in tumours growing in bone [63]. Importantly, high-resolution analysis of tumours shows prostate cancer bone metastasis have an actionable immunosuppressive microenvironment [46]. Our study reveals Siglec-sialoglycan interaction as a critical player in how immune cells support malignant prostate cells within the bone marrow and identifies a vulnerability amenable to therapeutic intervention.

While initial interrogation of the Siglec-sialoglycan axis in cancer focused on blocking Siglec receptors [16], it has been suggested that, due to redundancy, targeting a single Siglec or one or more of its ligands, may not be an effective mean of inhibiting this pathway. Engineered enzymes to strip terminal sialic acids from the glycans that cover the surface of cancer and immune cells could overcome Siglec redundancy to effectively inhibit multiple Siglec receptors. Bisialidase enzymes have been used therapeutically in mouse models to halt the growth of breast, melanoma and colon cancers [34]. Our findings indicate that this approach is also therapeutically relevant for prostate cancer, and specifically for prostate-derived tumours growing in bone, which represent an incurable major clinical burden. In clinical studies, E-602 is currently given as weekly intravenous injections, and in 95 subjects treated to date, E-602 has been well tolerated at doses up to 30 mg/kg (NCT05259696) [77, 78]. However, an ideal way to further improve the safety and efficacy of this therapeutic strategy is the targeted desialylation of cancer cells. Targeted sialidase therapy, with a ligand that binds to HER2 receptors has demonstrated efficacy to treat breast cancer [35, 79]. We propose that a similar therapeutic strategy could be applied to leverage enzymatic sialoglycan degradation to specifically target prostate cancer cells. Palleon have already developed E-688, a B7H3-targeted sialidase [80]. Excitingly, the immune checkpoint protein B7H3 is highly expressed on the surface of the most lethal prostate tumours [81], and we plan to evaluate E-688 in future studies for prostate cancer therapy.

In conclusion, our study reveals Siglec-engaging immunosuppressive sialoglycans are upregulated in prostate tumours and correlate with bone metastasis and reduced survival times in patients. Our findings show sialoglycan-Siglec biology is important in the tumour microenvironment of bone metastatic prostate cancer. Furthermore, we demonstrate that therapeutic targeting of tumour sialylation increases immune cell infiltration and can effectively impede the growth of prostate cancer in bone. Together, these results provide strong rationale for the further clinical development of sialoglycan-Siglec targeting therapies for bone metastatic prostate cancer and their investigation in combination with existing immunotherapy and anti-androgen therapies.

## Supplementary information

Supplementary Figures

Supplementary Figure Legends

Arrive checklist

## Data Availability

The authors confirm that the data supporting the findings of this study are available within the article and its supplementary materials.
