# Supplementary material for: Targeting Siglec-engaging immunosuppressive sialoglycans to suppress prostate cancer bone metastasis
Source: Br J Cancer. 2026 Jul 13;135(7):1164–77. doi: 10.1038/s41416-026-03544-5 (PMC13578327; doi:10.1038/s41416-026-03544-5)

Supplementary Figure 1

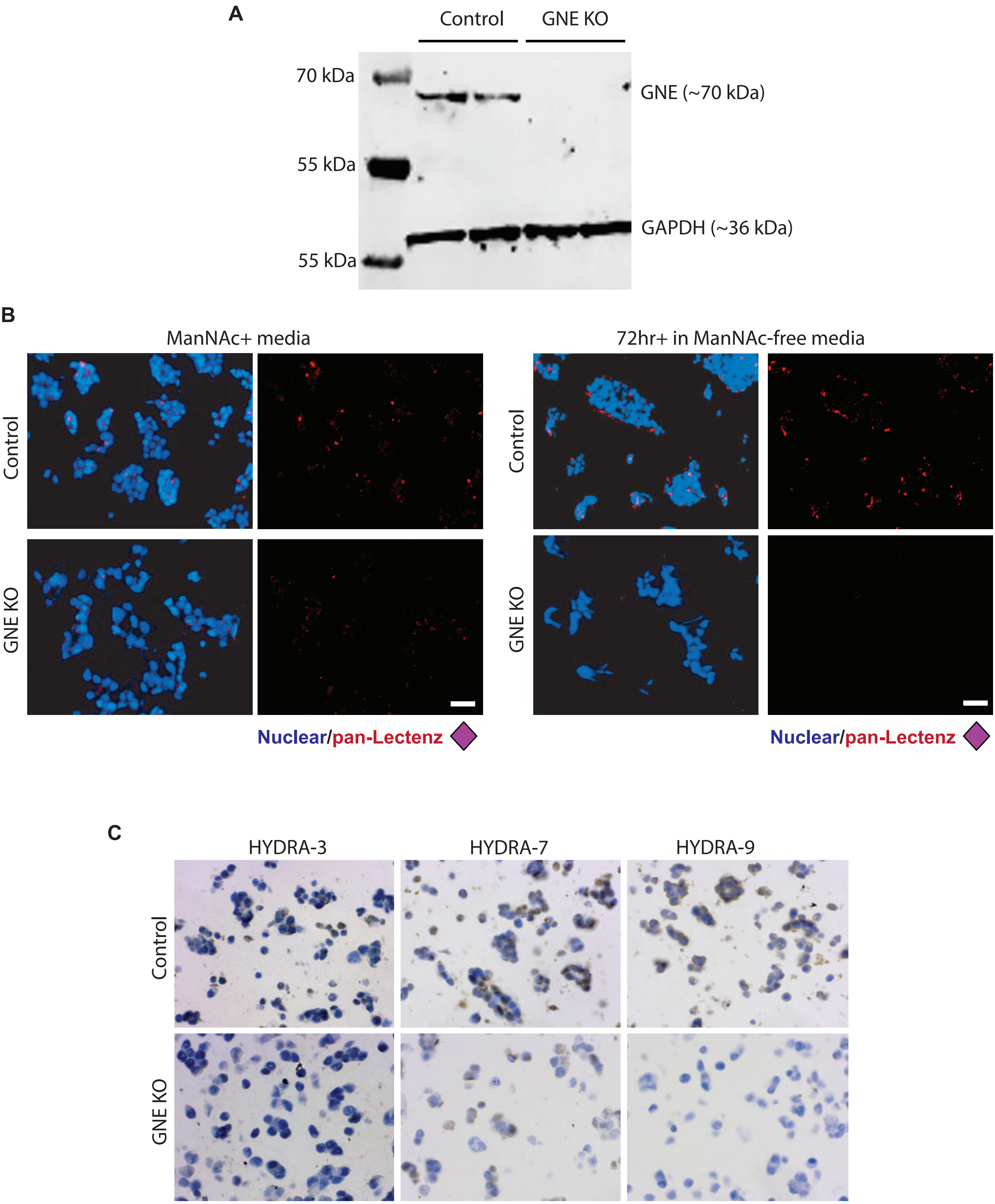

Supplementary Figure 2

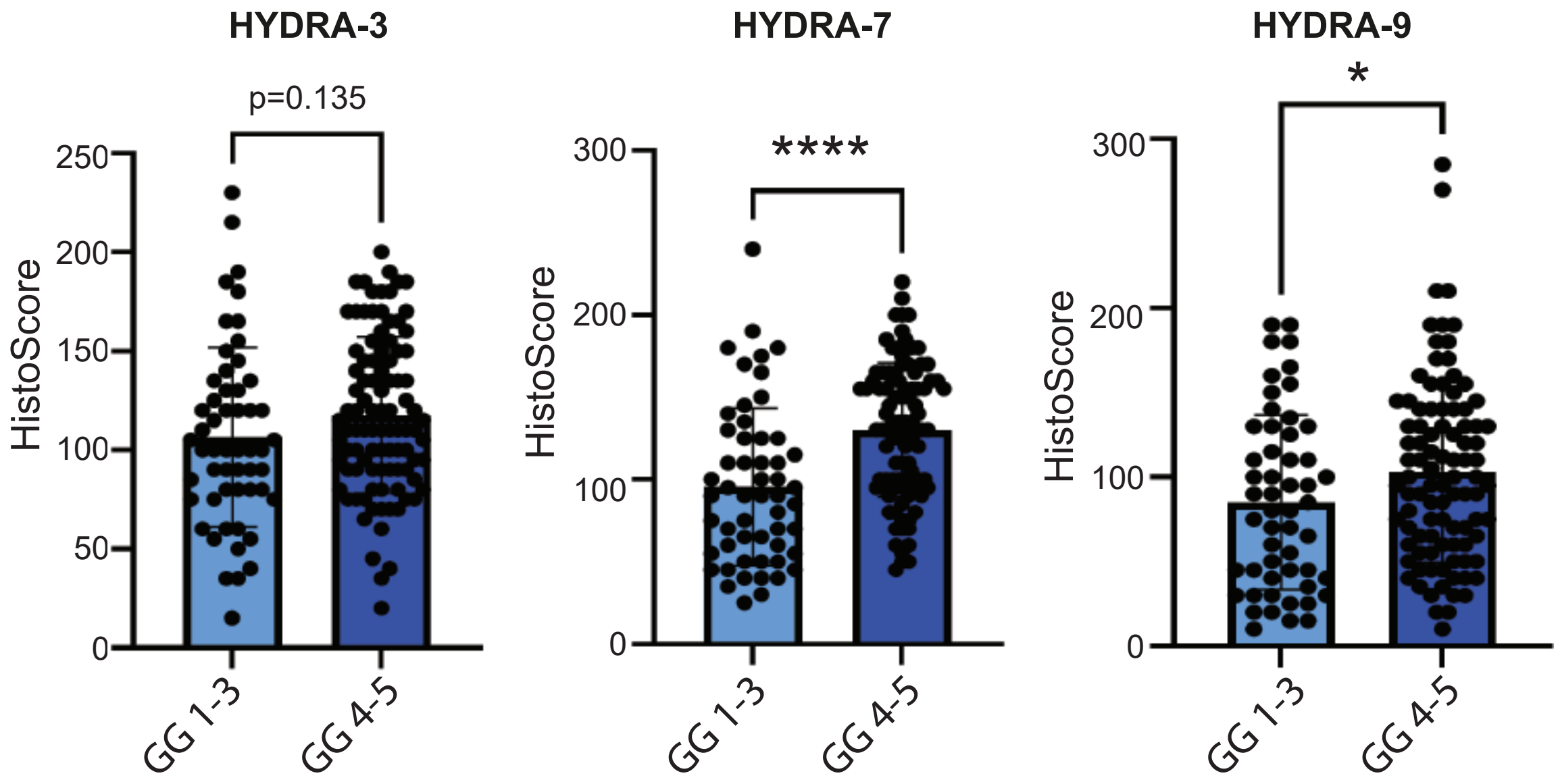

Supplementary Figure 3

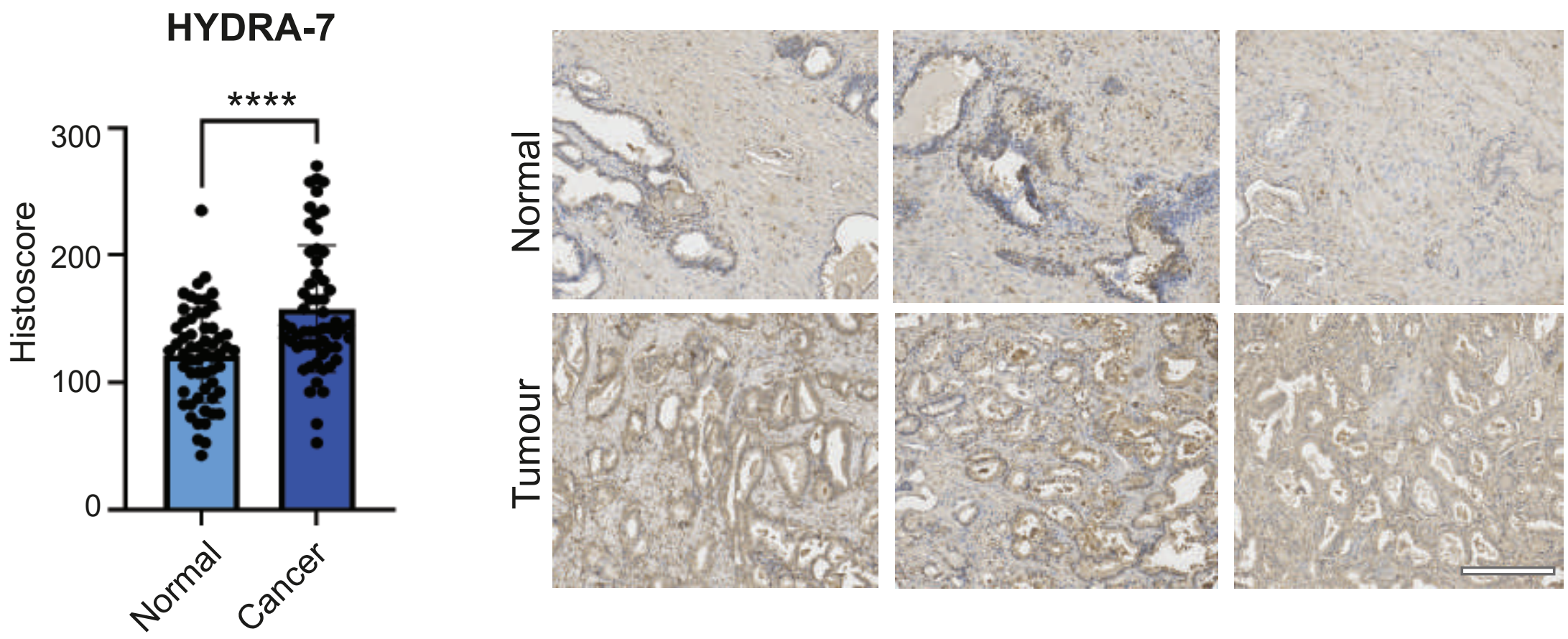

Supplementary Figure 4

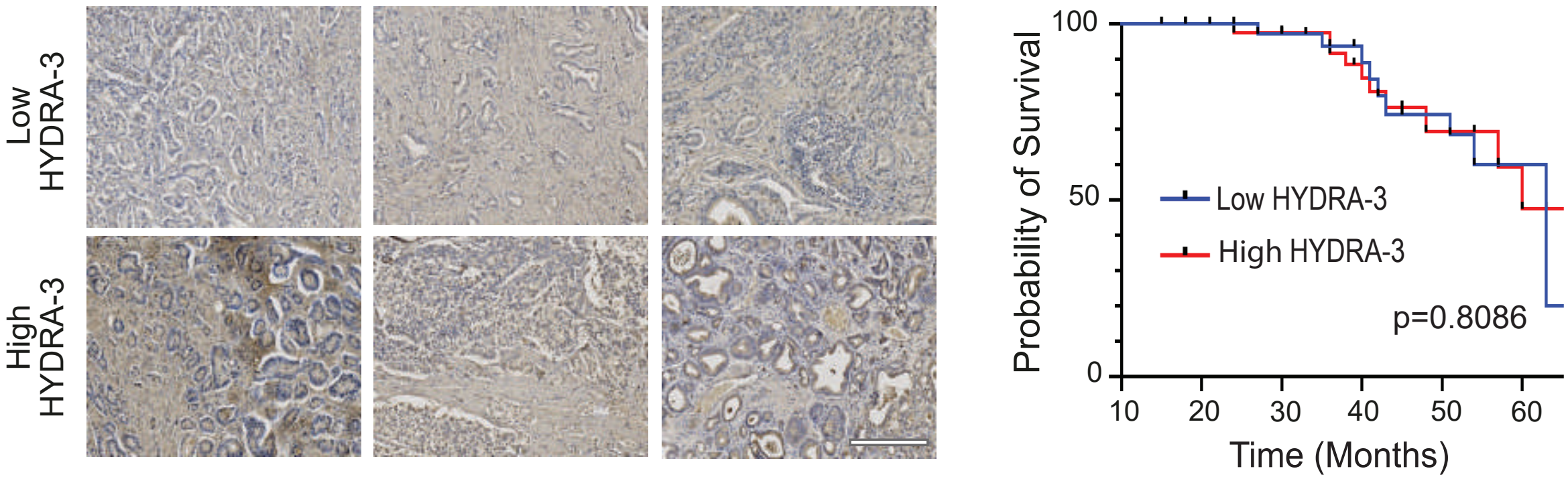

Supplementary Figure 5

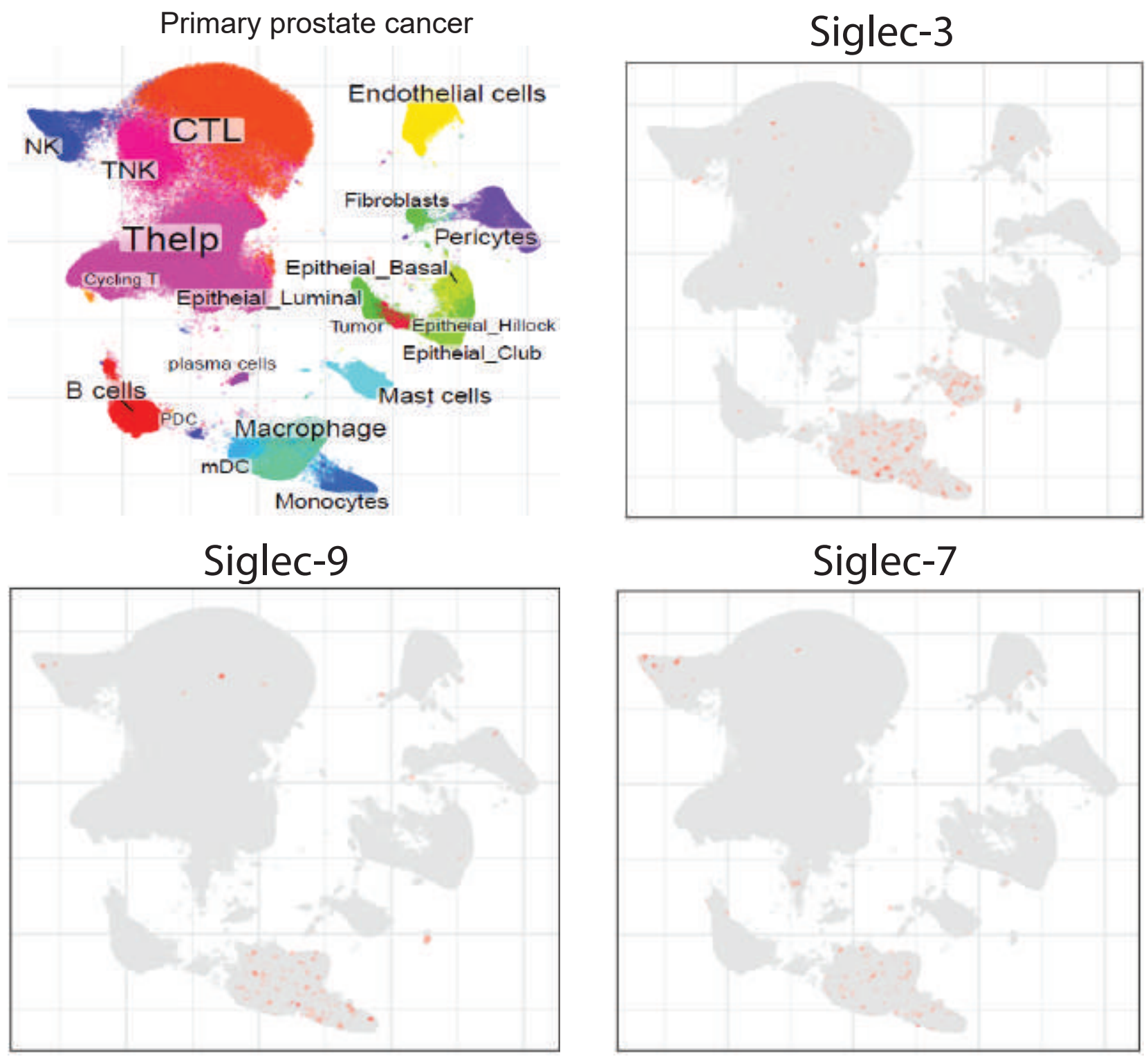

Supplementary Figure 6

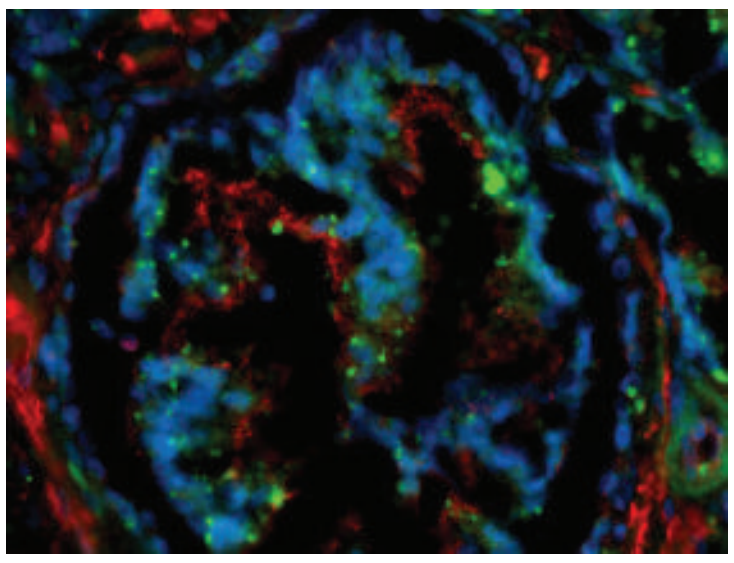

Nuclear / Siglec-3 / CD14

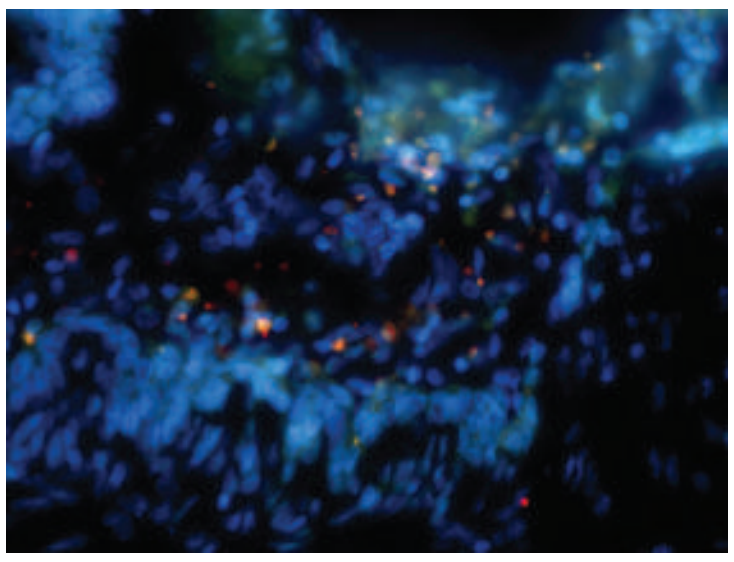

Nuclear / Siglec-3 / AMACR

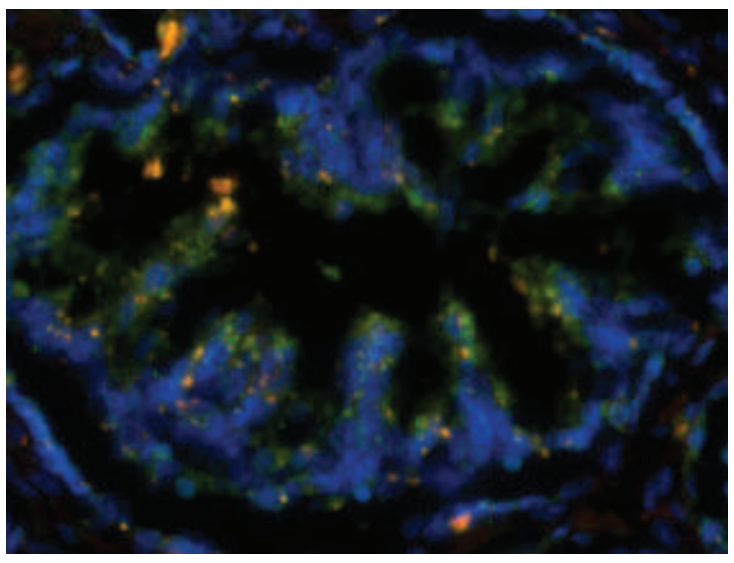

Nuclear / Siglec-7 / CD14

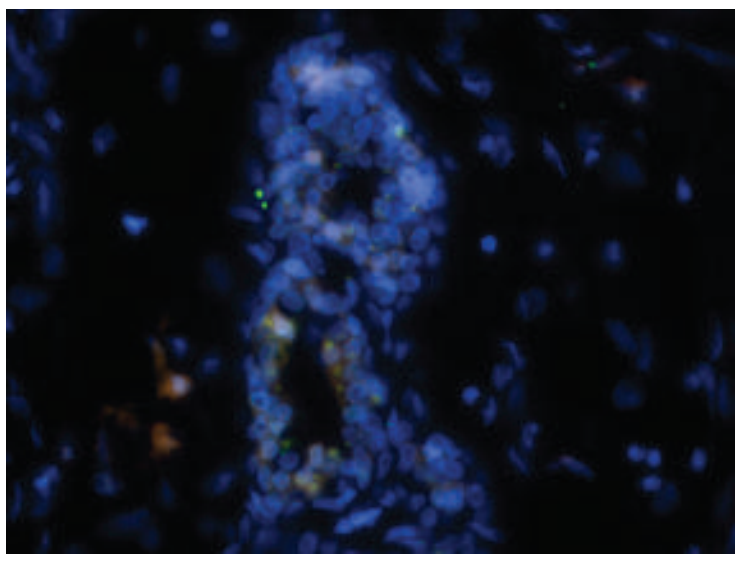

Nuclear / Siglec-7 / AMACR

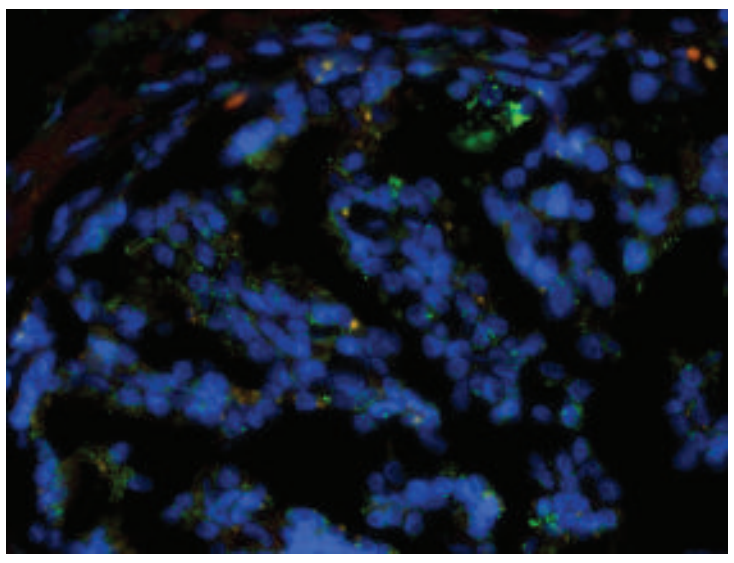

Nuclear / Siglec-9 / CD14

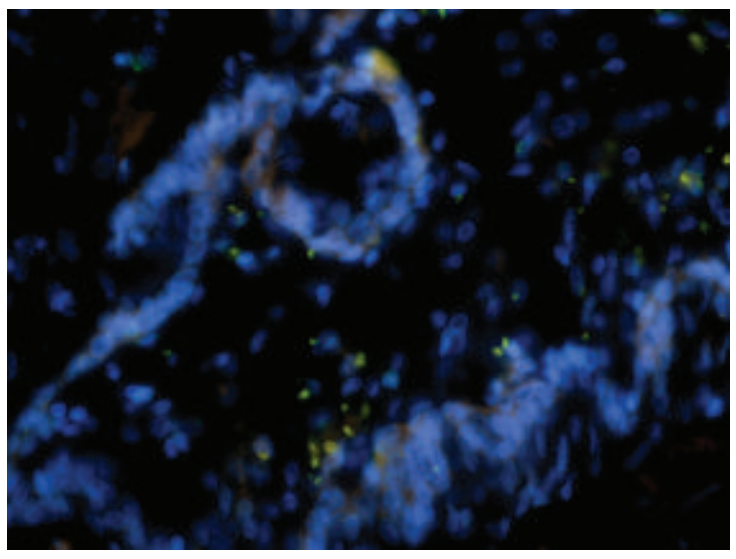

Nuclear / Siglec-9 / AMACR

Supplementary Figure 7

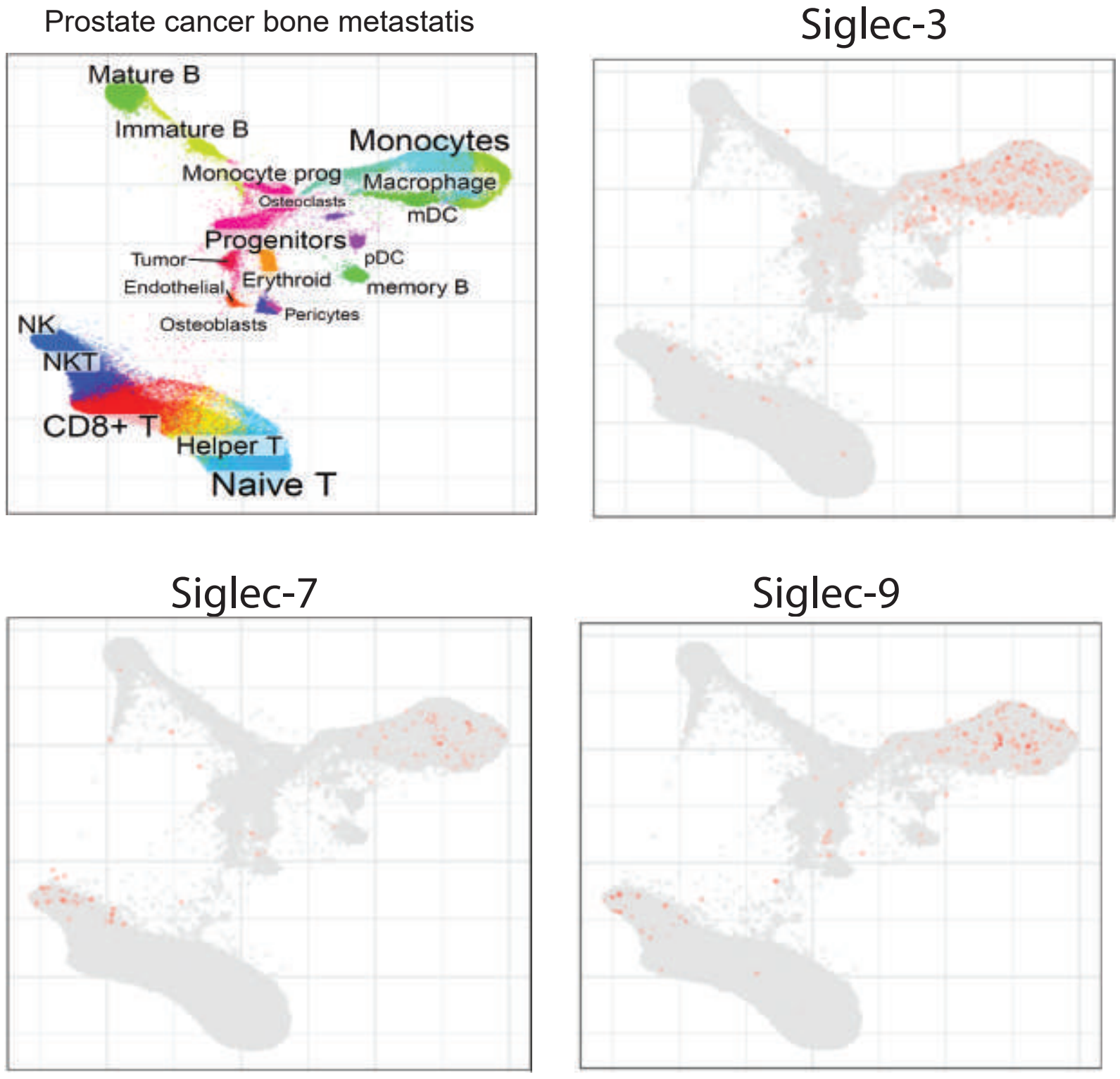

Supplementary Figure 8

Prostate-derived tumours growing in bone

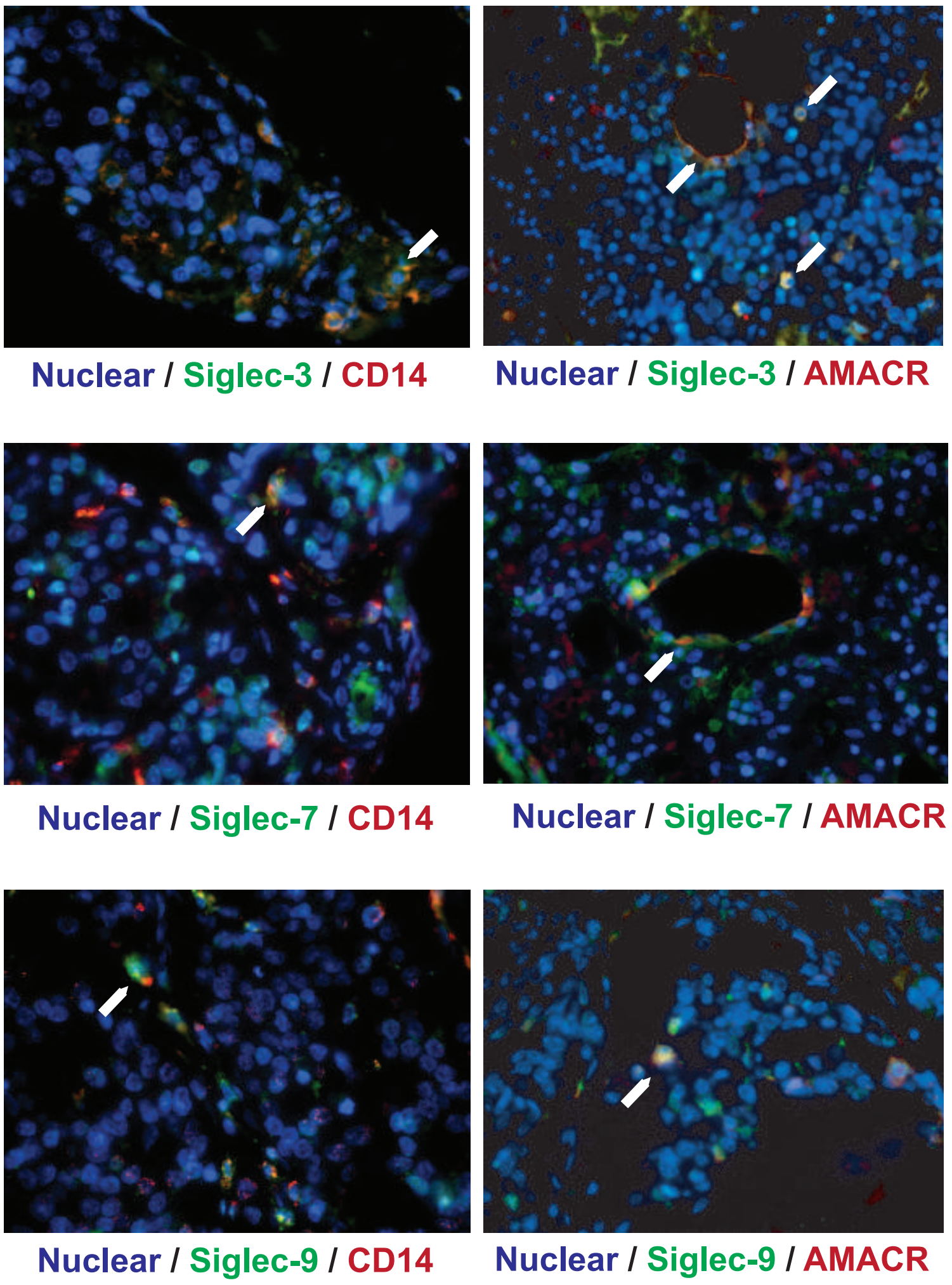

Supplementary Figure 9

A

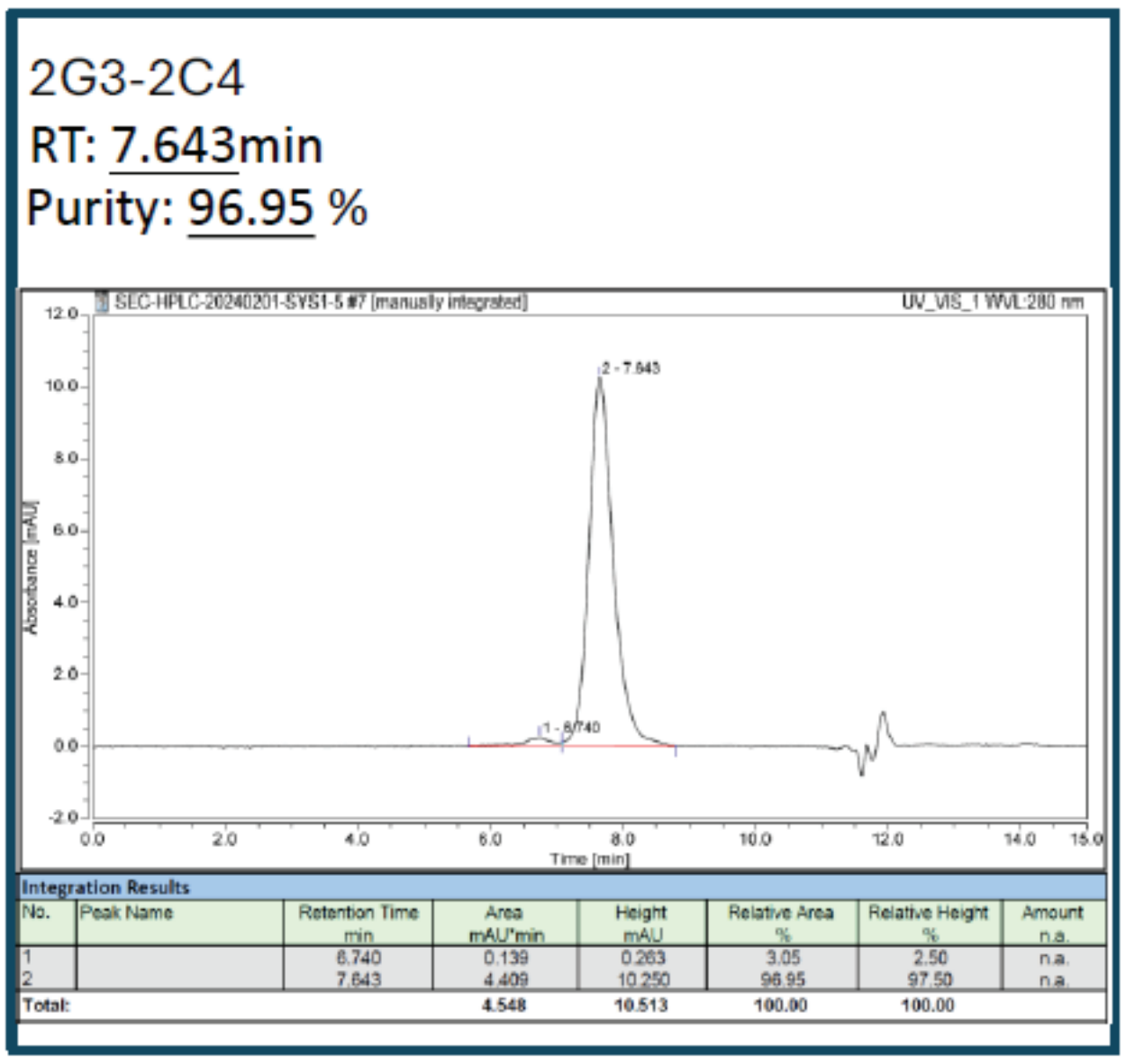

No.	Peak Name	Retention Time min	Area mAU*min	Height mAU	Relative Area %	Relative Height %	Amount n.a.
1		6.740	0.139	0.263	3.05	2.50	n.a.
2		7.643	4.409	10.250	96.95	97.50	n.a.
Total:			4.548	10.513	100.00	100.00	

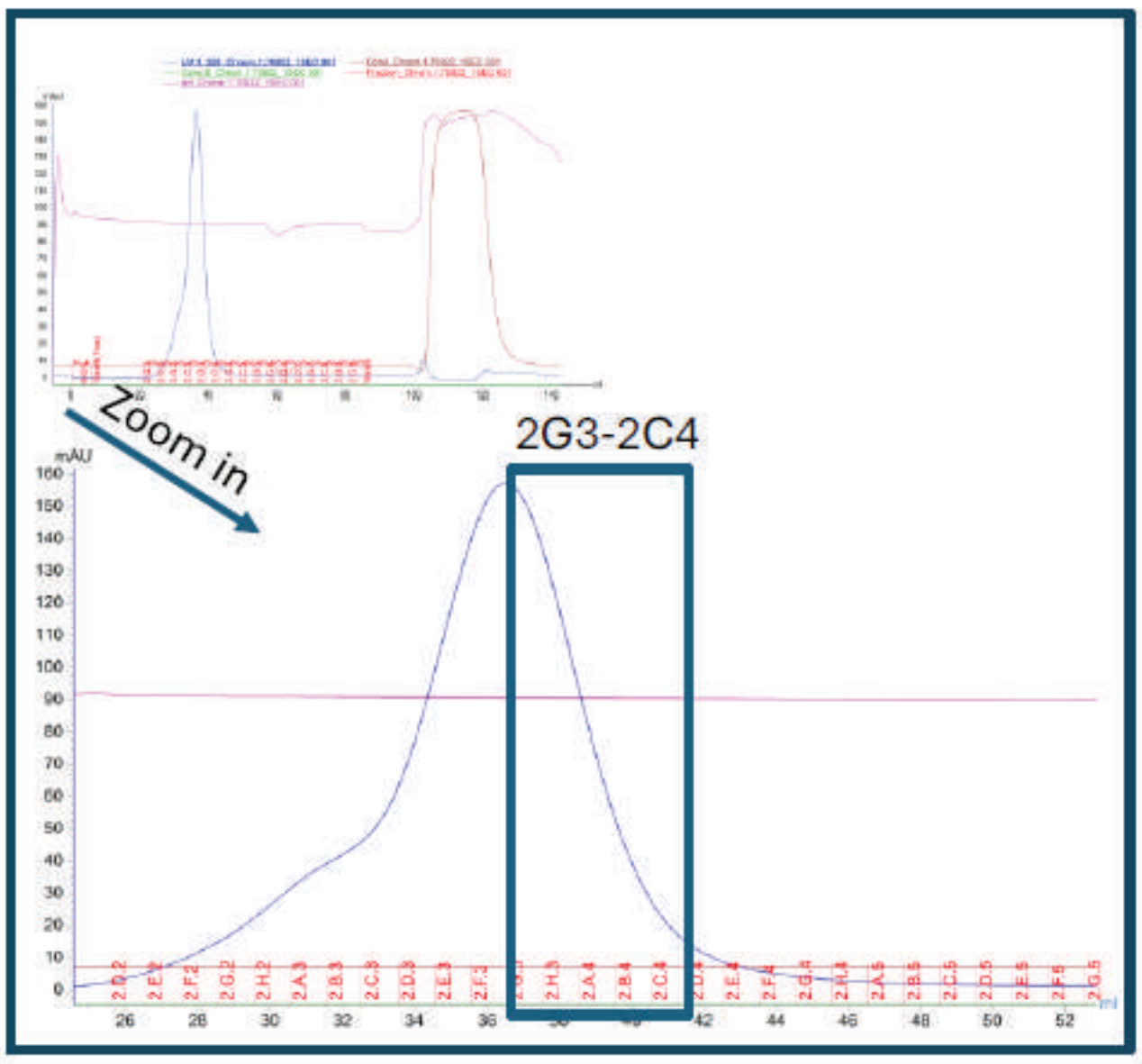

B

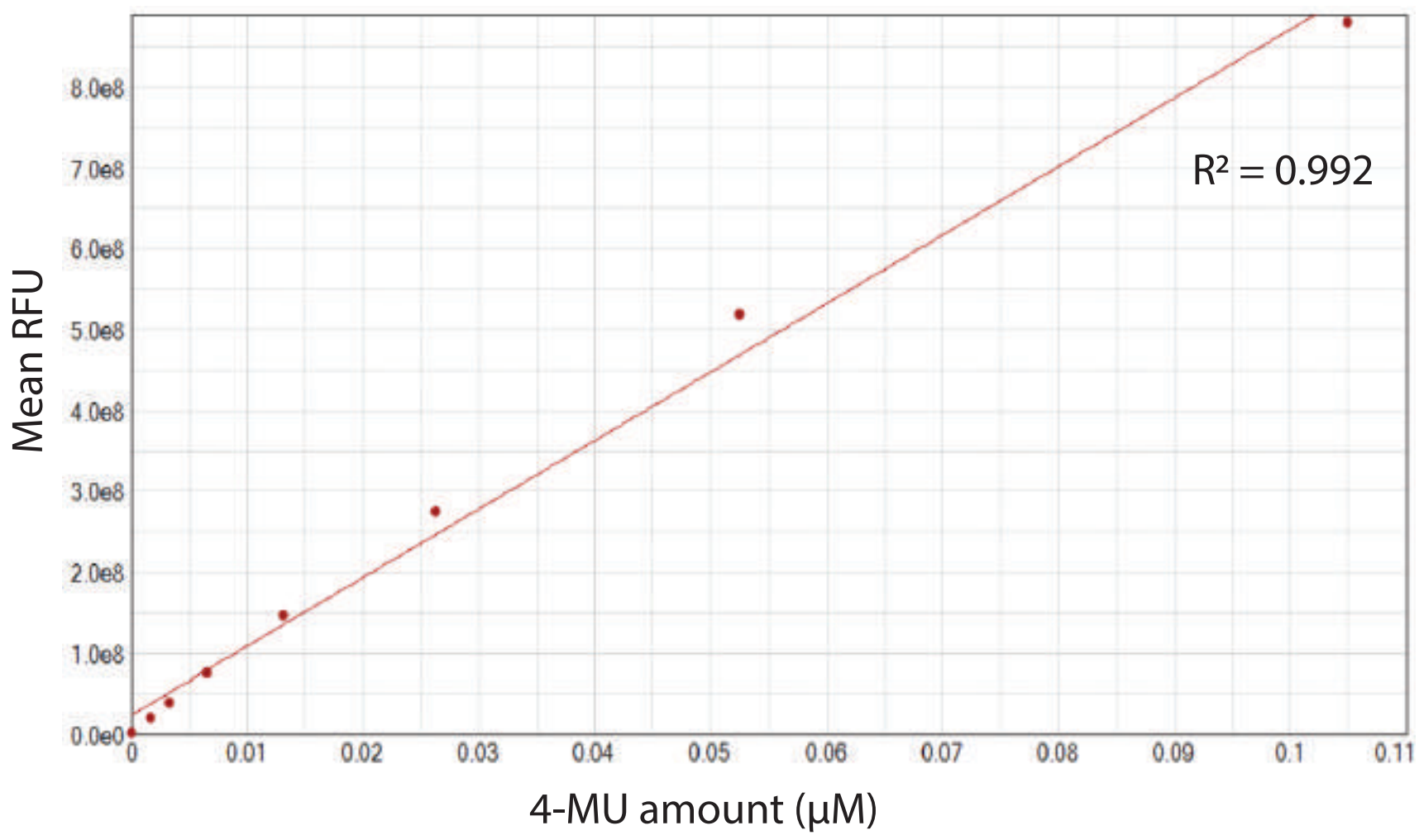

SC-H

Sample	Well	Concentration µM	micromole	Values	MeanValue	CV%	recovery%
01	A3<br>A4	700.000	0.105000	863360128.000<br>873238080.000	868299104.000	0.804	94.929

SC-M

Sample	Well	Concentration µM	micromole	Values	MeanValue	CV%	recovery%
01	B3<br>B4	175.000	0.026250	258950976.000<br>255137312.000	257044144.000	1.049	104.885

Control

Sample	Well	Values	MeanValue	CV%
01	C3<br>C4	5497108.000<br>5401836.000	5449472.000	1.236

C

Sample ID	Reaction time	Conc.	Normalized RFU value within SC01-SC05 (Y/N)	Catalyzed substrate(4-MU) (µmol)	Enzymatic activity (mU/mg)
E-612 (lot WBP70922_1)	28 min	0.06mg/ml	Yes	0.040248	479

Supplementary Figure 10

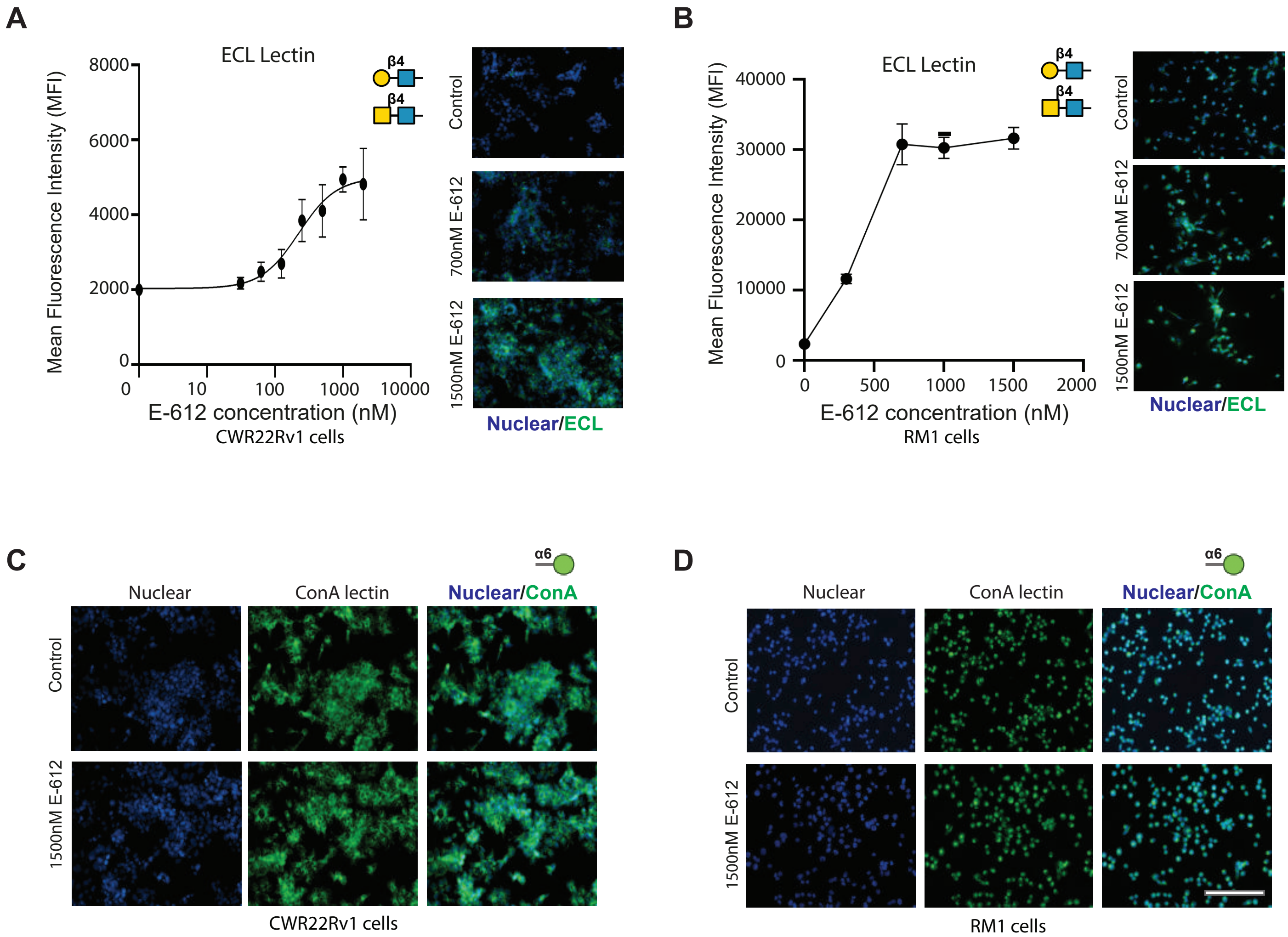

Supplementary Figure 11

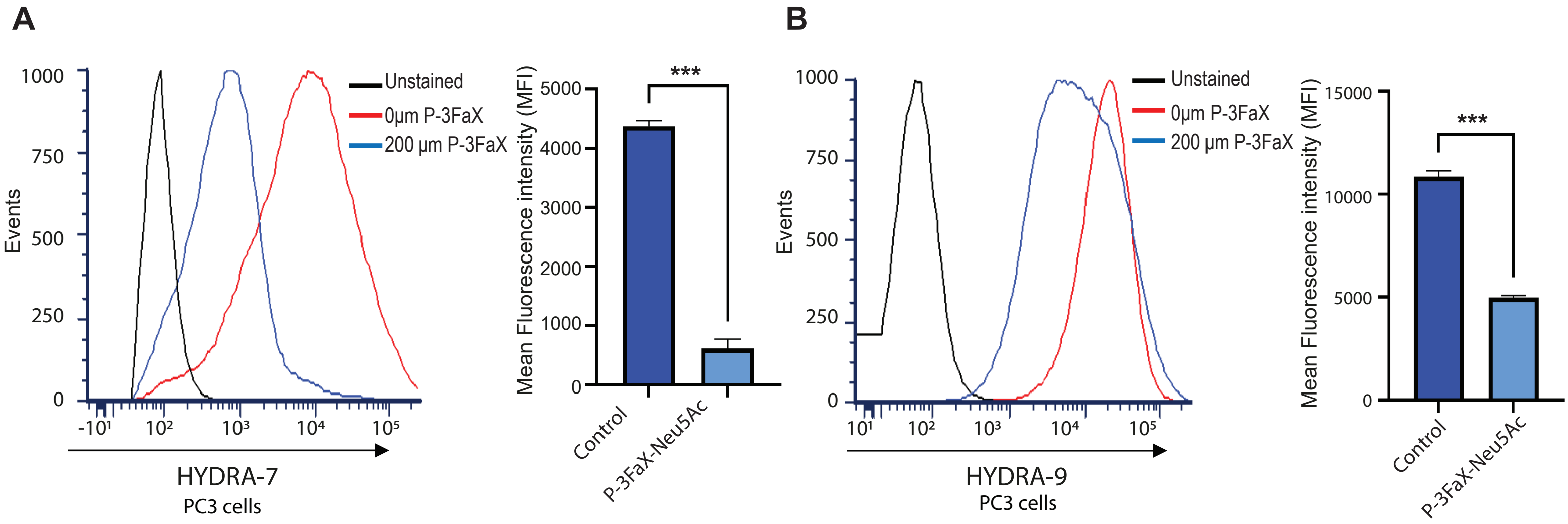

Supplement: Supplementary file 1 [file 41416_2026_3544_MOESM1_ESM.pdf]
