# Supplementary material for: Targeting Siglec-engaging immunosuppressive sialoglycans to suppress prostate cancer bone metastasis
Source: Br J Cancer. 2026 Jul 13;135(7):1164–77. doi: 10.1038/s41416-026-03544-5 (PMC13578327; doi:10.1038/s41416-026-03544-5)
Supplement: Supplementary file 2 [file 41416_2026_3544_MOESM3_ESM.pdf]

NOTE: Please save this file locally before filling in the table, DO NOT work on the file within your internet browser as changes will not be saved. Adobe Acrobat Reader (available free here) is recommended for completion.

The ARRIVE guidelines 2.0: author checklist

The ARRIVE Essential 10

These items are the basic minimum to include in a manuscript. Without this information, readers and reviewers cannot assess the reliability of the findings.

Item		Recommendation	Section/line number, or reason for not reporting
**Study design**	1	For each experiment, provide brief details of study design including:	
		a. The groups being compared, including control groups. If no control group has been used, the rationale should be stated.	Mouse model section, page 1
		b. The experimental unit (e.g. a single animal, litter, or cage of animals).	Mouse model section, page 11
**Sample size**	2	a. Specify the exact number of experimental units allocated to each group, and the total number in each experiment. Also indicate the total number of animals used.	Mouse model section, page 11, line 352 and page 11, line 359 and Figure 6 legend.
		b. Explain how the sample size was decided. Provide details of any *a priori* sample size calculation, if done.	8-10 mice per experimental group was used in order to balance between reducing the number of mice needed and providing enough statistical power to generate robust data. Power calculations was based on our
**Inclusion and exclusion criteria**	3	a. Describe any criteria used for including and excluding animals (or experimental units) during the experiment, and data points during the analysis. Specify if these criteria were established *a priori*. If no criteria were set, state this explicitly.	No criteria for all experiments unless as statistically identified outliers
		b. For each experimental group, report any animals, experimental units or data points not included in the analysis and explain why. If there were no exclusions, state so.	No exclusion unless statistically identified outliers
		c. For each analysis, report the exact value of *n* in each experimental group.	Figure 6 legend
**Randomisation**	4	a. State whether randomisation was used to allocate experimental units to control and treatment groups. If done, provide the method used to generate the randomisation sequence.	Mouse model section, on page 11, line 353.
		b. Describe the strategy used to minimise potential confounders such as the order of treatments and measurements, or animal/cage location. If confounders were not controlled, state this explicitly.	Mouse model section, page 11, line 349 and 353.
**Blinding**	5	Describe who was aware of the group allocation at the different stages of the experiment (during the allocation, the conduct of the experiment, the outcome assessment, and the data analysis).	Mouse model section,page 11. MF allocated the groups and performed imaging. NW performed injections and analysed data but was kept blinded to the allocation.
**Outcome measures**	6	a. Clearly define all outcome measures assessed (e.g. cell death, molecular markers, or behavioural changes).	Mouse model section in page 11 and Result section 6, page 16.
		b. For hypothesis-testing studies, specify the primary outcome measure, i.e. the outcome measure that was used to determine the sample size.	N/A
**Statistical methods**	7	a. Provide details of the statistical methods used for each analysis, including software used.	Statistical analysis section Page 12 and figure legends
		b. Describe any methods used to assess whether the data met the assumptions of the statistical approach, and what was done if the assumptions were not met.	In figure legends
**Experimental animals**	8	a. Provide species-appropriate details of the animals used, including species, strain and substrain, sex, age or developmental stage, and, if relevant, weight.	Mouse model section,page 11
		b. Provide further relevant information on the provenance of animals, health/immune status, genetic modification status, genotype, and any previous procedures.	Mouse model section,page 11
**Experimental procedures**	9	For each experimental group, including controls, describe the procedures in enough detail to allow others to replicate them, including:	
		a. What was done, how it was done and what was used.	Mouse model section,page 11
		b. When and how often.	Mouse model section,page 11
		c. Where (including detail of any acclimatisation periods).	Mouse model section,page 11
		d. Why (provide rationale for procedures).	Mouse model section,page 11
**Results**	10	For each experiment conducted, including independent replications, report:	
		a. Summary/descriptive statistics for each experimental group, with a measure of variability where applicable (e.g. mean and SD, or median and range).	Statistical analysis section Page 12 for general information and further statistical methods were detailed in relevant result section and figure legends
		b. If applicable, the effect size with a confidence interval.	N/A

The Recommended Set

These items complement the Essential 10 and add important context to the study. Reporting the items in both sets represents best practice.

Item		Recommendation	Section/line number, or reason for not reporting
**Abstract**	11	Provide an accurate summary of the research objectives, animal species, strain and sex, key methods, principal findings, and study conclusions.	Page 2 and11
**Background**	12	a. Include sufficient scientific background to understand the rationale and context for the study, and explain the experimental approach.	Page 5
		b. Explain how the animal species and model used address the scientific objectives and, where appropriate, the relevance to human biology.	Page 5, line 127-128.
**Objectives**	13	Clearly describe the research question, research objectives and, where appropriate, specific hypotheses being tested.	Page 5, last paragraph
**Ethical statement**	14	Provide the name of the ethical review committee or equivalent that has approved the use of animals in this study, and any relevant licence or protocol numbers (if applicable). If ethical approval was not sought or granted, provide a justification.	Page 11, last paragraph
**Housing and husbandry**	15	Provide details of housing and husbandry conditions, including any environmental enrichment.	Page 11, second paragraph
**Animal care and monitoring**	16	a. Describe any interventions or steps taken in the experimental protocols to reduce pain, suffering and distress.	Page 11, line 354 and 366.
		b. Report any expected or unexpected adverse events.	Page 11, line 354
		c. Describe the humane endpoints established for the study, the signs that were monitored and the frequency of monitoring. If the study did not have humane endpoints, state this.	Page 11, line 354 and 366
**Interpretation/ scientific implications**	17	a. Interpret the results, taking into account the study objectives and hypotheses, current theory and other relevant studies in the literature.	Result subsection 6, page 16-17.
		b. Comment on the study limitations including potential sources of bias, limitations of the animal model, and imprecision associated with the results.	Page 16, 1st paragraph
**Generalisability/ translation**	18	Comment on whether, and how, the findings of this study are likely to generalise to other species or experimental conditions, including any relevance to human biology (where appropriate).	Page 11, 2nd paragraph
**Protocol registration**	19	Provide a statement indicating whether a protocol (including the research question, key design features, and analysis plan) was prepared before the study, and if and where this protocol was registered.	Page 11. Protocols were all prepared and reviewed by NVS before the studies and logged with local local Research Ethics Committees of the University of Sheffield under Home Office project licence (PP3267943)
**Data access**	20	Provide a statement describing if and where study data are available.	Page 21, Data sharing statement Section
**Declaration of interests**	21	a. Declare any potential conflicts of interest, including financial and non-financial. If none exist, this should be stated.	Page 21 Declaration of Interests'
		b. List all funding sources (including grant identifier) and the role of the funder(s) in the design, analysis and reporting of the study.	Funding section, page 21
