# Supplementary material for: Targeting Siglec-engaging immunosuppressive sialoglycans to suppress prostate cancer bone metastasis
Source: Br J Cancer. 2026 Jul 13;135(7):1164–77. doi: 10.1038/s41416-026-03544-5 (PMC13578327; doi:10.1038/s41416-026-03544-5)
Supplement: Supplementary file 3 [file 41416_2026_3544_MOESM2_ESM.docx]

**Supplementary Figure 1. Validation of HYDRA immunohistochemistry for the detection of immunosuppressive sialoglycans in prostate cancer tissues.** (**A**) Detection of GNE using western blotting confirms loss of GNE protein in CWR22Rv1 cells with CRISPR-cas9 mediated knockout of GNE. (**B**) Immunofluorescence analysis shows CWR22Rv1 cells with knockout of GNE have reduced levels of sialic acid (detected using pan-specific Lectenz). (**C**) HYDRA-immunohistochemistry analysis of Siglec-3, -7 and -9 ligands in control and GNE knockout CWR22Rv1 FFPE cell pellets. Confirming the specificity of the HYDRA reagents for detection of sialoglycans in prostate cancer cells, the levels of all three ligands are reduced in GNE knockout pellets compared to control pellets.

**Supplementary Figure 2.** Sialoglycans that engage Siglec-7 and -9 are increased in high Gleason grade prostate cancer. HYDRA immunohistochemistry analysis of the 96 case TMA shown in Figure 1B showed higher expression of Siglec-7 ligands (p<0.0001) and Siglec-9 ligands (p=0.0437) in prostate tumours (Gleason grade 4-5) compared to lower grade prostate tumours (Gleason grade 1-3).

**Supplementary Figure 3.** HYDRA immunohistochemistry analysis of Siglec-7 ligands in TMA cohort 6 [41]. Siglec-7 engaging sialoglycans are significantly upregulated in primary prostate tissue compared to matched normal tissue (n=60, paired t test, p<0.0001).

**Supplementary Figure 4.** Analysis of Siglec-3 ligands in a 100-case prostate cancer TMA (TMA cohort 7) using HYDRA-3 immunohistochemistry. Stratification of patients based on high and low Siglec-3 ligand levels shows patients with high HYDRA-3 levels (defined as the top 50^th^ percentile of expression) have no significant difference in survival rates compared to patients with low HYDRA-3 levels (defined as the bottom 50^th^ percentile of expression) (n=100, Kaplan-Meier regression model, p= 0.8844).

**Supplementary Figure 5.** UMAP map of previously published single cell RNA-sequencing data from primary prostate tumours [46] showing individual *Siglec* expression levels across different cell types.

**Supplementary Figure 6.** **Siglec receptors are expressed by infiltrating immune cells in primary prostate cancer tumours.** Dual immunofluorescence analysis of Siglec receptors and immune cell markers in independent primary prostate tumour tissue samples. Siglec-3, -7, and -9 are co-expressed with CD14 (a myeloid marker) in primary prostate tumours. Siglec receptors co-localised with α-methylacyl-CoA racemase (AMACR) confirming their expression within cancerous tissues. Scale bar is 20 µm.

**Supplementary Figure 7. UMAP map of previously published single cell RNA-sequencing data from prostate cancer bone metastatic tissues** [45] showing individual *Siglec* expression levels across different cell types.

**Supplementary Figure 8. Siglec receptors are expressed by infiltrating immune cells in prostate-derived tumours growing in bone.** Dual immunofluorescence analysis of Siglec receptors and immune cell markers in independent prostate cancer bone metastasis tissue samples. Siglec-3 (CD33), Siglec-7, and Siglec-9, are co-expressed with CD14 (a myeloid marker). Siglec receptors co-localised with α-methylacyl-CoA racemase (AMACR) confirming their expression within cancerous tissues. Scale bar is 20 µm.

**Supplementary Figure 9. Characterisation, biochemical validation, and purity assessment of the engineered human Neu2 sialidase–Fc fusion protein (E-612**). (**A**) Size Exclusion Chromatography (SEC-HPLC) purity test shows the SED purity of E-612 is 96.95% monomer. (**B**) 4-MU standard curve and assay suitability passed with qualified SC01-SC05, SC-H and SC-M recovery (within 75%-125%). (**C**) Enzyme activity for E-612 was 479 mU/mg (using the 4-MU-Neu5Ac substrate).

**Supplementary Figure 10. E-612 bisialidase can effectively strip Siglec ligands from prostate cancer cells.** (**A,B**) Lectin flow cytometry and lectin immunofluorescence assays show CWR22Rv1 and RM1 cells treated with E-612 for 24 hours have increased levels of binding to Erythrina Cristagalli Lectin (ECL) (which binds terminal galactose or LacNAc epitopes [54] uncovered by the removal of sialic acids). (**C**) Staining with ConA lectin (which recognises glycans containing α-D-mannose [54] confirmed the sialic acid specificity of these changes.

**Supplementary Figure 11. Validation of HYDRA flow cytometry to detect Siglec ligands on prostate cancer cells.** Flow cytometry analysis of PC3 prostate cancer cells treated with 200 μM of the sialyltransferase inhibitor P-3FaX-Neu5Ac for 72 hours. Treatment with P-3FaX-Neu5Ac (which we have previously shown inhibits sialylated glycans in this cell line [25]) significantly reduces binding of both HYDRA-7 (Welch’s t test, P=0.0001) and HYDRA-9 (Welch’s t test, P=0.0008).
